# Macrophage FTO deficiency accelerates atherosclerosis via PACS2-mediated activation of the PPARγ lipid signaling pathway

**DOI:** 10.1186/s12967-026-08076-3

**Published:** 2026-03-31

**Authors:** Jie Ouyang, Zishun Zhan, Haijiao Long, Shuhua Chen, Hong Xiang, Baiyi Tang, Quanjun Liu, Shiying Qin, Ye Tao, Alex F. Chen, Hongwei Lu

**Affiliations:** 1 Center for Experimental Medical Research, The Third Xiangya Hospital of Central South University, Changsha, Hunan China; 2 Department of Cardiology, The Third Xiangya Hospital of Central South University, Changsha, Hunan China; 3 Department of Biochemistry, School of Life Sciences of Central South University, Changsha, Hunan China; 4 Intensive Care Unit, Yueyang Central Hospital, Yueyang, Hunan China; 5 The Institute for Cardiovascular Development and Regenerative Medicine, Xinhua Hospital Affiliated with Shanghai Jiaotong University School of Medicine, Shanghai, China

**Keywords:** Atherosclerosis, FTO, PACS2, PPARγ, Macrophage

## Abstract

**Background:**

Atherosclerosis, a leading cause of cardiovascular disease, is driven by abnormal lipid accumulation in arterial walls. However, the underlying molecular mechanisms remain incompletely understood. This study investigated the role of the macrophage-specific fat mass and obesity-associated gene (FTO) in atherogenesis.

**Methods:**

We used an adeno-associated virus serotype 9 (AAV9) vector under the control of the F4/80 promoter to overexpress FTO in macrophages. The functional roles of FTO and its molecular interactions were investigated through Western blotting, RT-qPCR, coimmunoprecipitation, immunofluorescence, and MeRIP-qPCR assays.

**Results:**

FTO expression was shown to be specifically reduced in macrophages. In primary peritoneal macrophages and RAW264.7 cells, reduced FTO expression increased lipid uptake and deposition. Overexpressing FTO significantly reduced high-fat diet (HFD)-induced atherosclerotic plaque formation and lipid accumulation in vivo. Mechanistically, FTO deficiency increased the mRNA stability of phosphofurin acidic cluster sorting protein 2 (*PACS2*) in an N6-methyladenosine (m^6^A)-dependent manner, thereby leading to elevated PACS2 protein levels and subsequent activation of the PPARγ pathway. This in turn resulted in increased expression of CD36 and PLIN2, which promoted lipid uptake and lipid droplet formation, respectively.

**Conclusions:**

Our findings identify a novel macrophage FTO-PACS2-PPARγ regulatory axis that plays a key role in lipid dysregulation and atherogenesis, thus highlighting FTO as a potential therapeutic target for atherosclerosis and related cardiovascular diseases.

**Supplementary Information:**

The online version contains supplementary material available at 10.1186/s12967-026-08076-3.

## Background

Atherosclerosis (AS), a condition driven by lipid accumulation in the arterial wall and chronic inflammation, is a primary cause of cardiovascular events such as myocardial infarction and peripheral artery disease [1]. Among the multiple cell types involved in plaque formation, macrophages play a pivotal role. Through excessive uptake and deposition of lipids, these cells transform into foam cells, which are central to lesion formation and progression [2]. Macrophage apoptosis further promotes necrotic core formation, thereby increasing plaque vulnerability and the risk of rupture [3]. Despite advances in the understanding of the roles of macrophages in cardiovascular pathology, the molecular mechanisms underlying lipid handling and its dysregulation in AS remain incompletely understood.

N6-methyladenosine (m^6^A), the most abundant form of internal mRNA modification in eukaryotes, regulates posttranscriptional gene expression by influencing RNA splicing, stability, translation, and decay [4]. This reversible process, which is mediated by molecules termed writers, erasers, and readers, is involved in various stress responses and diseases. A growing body of evidence links m^6^A dysregulation to cardiovascular pathologies [5], including emerging roles for macrophage m^6^A methylation in AS [6]. Nonetheless, the mechanistic contributions of m^6^A to macrophage lipid metabolism in AS are still poorly defined.

The fat mass and obesity-associated protein (FTO), which is a key m^6^A demethylase, regulates mRNA methylation and influences metabolic and disease-related gene expression [7, 8]. Altered FTO function is associated with metabolic syndromes, including obesity and diabetes [9, 10], and has been implicated in cardiovascular conditions such as cardiac hypertrophy, congenital defects, and arrhythmias [11–13]. Interestingly, recent research suggests that FTO may suppress lipid uptake in macrophages and attenuate AS [14]. However, the precise m^6^A-dependent mechanism through which FTO modulates lipid metabolism in macrophages remains unclear. Moreover, although the OxLDL-induced PPARγ pathway is a well-established driver of foam cell formation [15], the potential interplay between FTO and this pathway has not been explored.

To address these questions, we constructed a macrophage-specific FTO-overexpressing *ApoE*^*−/−*^ model using an adeno-associated virus serotype 9 vector under the F4/80 promoter (AAV9-F4/80-oeFTO). We observed that macrophage-specific FTO overexpression mitigated atherosclerotic progression and reduced lipid accumulation in vivo. In *ApoE*^*−/−*^*PACS2*^*−/−*^ mice treated with the FTO inhibitor FB23-2, we demonstrated that FTO promotes AS by regulating PACS2. In vitro studies involving primary peritoneal macrophages (PMs) and RAW264.7 cells confirmed that FTO loss exacerbates OxLDL-induced lipid uptake and deposition, whereas its overexpression attenuates these processes. Mechanistically, MeRIP-qPCR revealed that FTO directly demethylates *PACS2* mRNA in an m^6^A-YTHDF2-dependent manner. FTO deficiency increased *PACS2* mRNA stability and protein expression, thereby activating the PPARγ signaling pathway and upregulating its downstream targets CD36 and PLIN2, which facilitate lipid uptake and droplet formation, respectively. Collectively, these results identify a novel FTO-PACS2-PPARγ axis as a key regulator of macrophage lipid metabolism in AS, thus highlighting FTO as a potential therapeutic target.

## Materials and methods

### Animal models

A total of 61 male mice were used in this study. *ApoE*^*−/−*^ mice on a C57BL/6J background were housed under specific pathogen-free (SPF) conditions at 25 °C and 50–60% humidity with a 12-hour light/dark cycle and provided ad libitum access to food and water. Based on the studies of others, male mice were selected to avoid the potential influence of estrogen on the formation of atherosclerotic plaques [16, 17]. Mice were group-housed at a density of six animals per cage in individually ventilated cages with corncob bedding and environmental enrichment. The animals were randomly assigned to experimental groups. All animal procedures were conducted in accordance with the National Institutes of Health Guide for the Care and Use of Laboratory Animals.

To generate macrophage-specific FTO-overexpressing mice, *ApoE*^*−/−*^ mice received tail vein injections of an adeno-associated virus serotype 9 vector driven by the F4/80 promoter (AAV9-F4/80-FTO; Hanbio, Shanghai, China). Control mice were injected with AAV9-F4/80-Null (designated as AAV-Null and AAV-FTO, respectively). To evaluate the role of PACS2 in FTO-mediated m⁶A regulation during atherosclerosis, we used *ApoE*^*−/−*^*PACS2*^*−/−*^ mice on a C57BL/6J background (which had been previously generated by our group [18]). The FTO inhibitor FB23-2 (20 mg/kg, HY-127103, MCE, USA) was dissolved in 2% DMSO and administered via intraperitoneal injection. Control animals received vehicle only. The animals were divided into the following experimental groups: the *ApoE*^*−/−*^ + negative control (NC), *ApoE*^*−/−*^ + FB23-2, *ApoE*^*−/−*^*PACS2*^*−/−*^ + NC, and *ApoE*^*−/−*^*PACS2*^*−/−*^ + FB23-2 groups. Atherosclerosis was induced by feeding 6–8-week-old mice a high-fat diet (HFD; D12108C, Research Diets, New Brunswick, USA) for 12 weeks. The composition of the HFD was 40% kcal from fat, 40% from carbohydrates, and 20% from protein, with 1.25% (w/w) cholesterol.

At the experimental endpoint, the mice were anesthetized with 1% pentobarbital (50 mg/kg, i.p.) and transcardially perfused with PBS. Hearts and aortas were harvested and either fixed in 4% paraformaldehyde or snap-frozen in liquid nitrogen for subsequent analysis. Mice were euthanized under deep anesthesia, and animal carcasses were disposed of by the Department of Laboratory Animals of Central South University following approved biosafety regulations.

### Quantification of lesion size

The aortic root was embedded in optimal cutting temperature (OCT) compound following fixation and sucrose dehydration. Serial 10 μm-thick frozen sections were stained with Oil Red O or hematoxylin and eosin (H&E). The atherosclerotic lesion area was quantified with ImageJ software (NIH, USA).

### Immunofluorescence staining

Frozen sections of the aortic sinus were fixed in 4% paraformaldehyde, permeabilized with 0.3% Triton X-100, and blocked with 5% bovine serum albumin (BSA). Sections were incubated overnight at 4 °C with primary antibodies against F4/80 (sc-377009, Santa Cruz Biotechnology, Dallas, USA), FTO (ab280081, Abcam, Cambridge, UK), PACS2 (30172-1-AP, Proteintech, Wuhan, China), CD36 (85127-6-RR, Proteintech), or PLIN2 (15294-1-AP, Proteintech), followed by incubation with the appropriate fluorescent secondary antibodies. The nuclei were counterstained with DAPI. Images were captured by using a Zeiss Axio Vert.A1 microscope.

### Cell culture

PMs and the RAW264.7 cell line were used as in vitro models, which is consistent with previously established approaches for studying macrophages in atherosclerosis [19].

PMs were isolated from mice that were intraperitoneally injected with 4% thioglycollate (Sigma-Aldrich, St. Louis, USA). Three days post-injection, the mice were euthanized, and the peritoneal cavities were lavaged with RPMI-1640 medium (Thermo Fisher Scientific, Waltham, USA). The collected cells were centrifuged and resuspended in RPMI-1640 supplemented with 10% fetal bovine serum (ScienCell, Carlsbad, USA). RAW264.7 cells, which were obtained from the American Type Culture Collection (ATCC; Rockville, USA), were maintained in Dulbecco’s modified Eagle’s medium (DMEM) (ScienCell) supplemented with 10% fetal bovine serum. Both cell types were cultured at 37 °C in a 5% CO₂ atmosphere. For atherosclerosis-related stimulation, cells were treated with 100 µg/mL oxidized LDL (Ox-LDL) (Yiyuan Biotechnologies, Guangzhou, China).

### Gene transfection

The coding sequences of FTO and PACS2 were cloned and inserted into the pcDNA3.1-3xFlag vector (Weizhen Biotech, Shanghai, China) to construct overexpression plasmids. For both plasmid and siRNA transfections, macrophages at approximately 80% confluence were transfected with Lipofectamine 3000 reagent (Thermo Fisher Scientific) in serum-free medium according to the manufacturer’s instructions. Transfection was performed with either 2 µg of plasmid or 50 nM siRNA targeting YTHDF2 (si-YTHDF2; GenePharma, Shanghai, China). The sequences of the siRNAs are listed in Table 1. After 8 h, the transfection medium was replaced with complete culture medium. Transfection efficiency was verified with RT-qPCR and Western blotting analysis.

**Table 1:** Small interfering RNA (siRNA) targeting YTHDF2

siRNA		Sense	Antisense
si-YTHDF2	CCGUUCCAUUAAGUAUAAUTT		AUUAUACUUAAUGGAACGGTT
Negative Control	UUCUCCGAACGUGUCACGUTT		ACGUGACACGUUCGGAGAATT

### Real-time quantitative polymerase chain reaction (RT-qPCR)

Total RNA was extracted with TRIzol reagent (Invitrogen, Carlsbad, USA). cDNA was synthesized with a reverse transcription kit (Toyobo, Osaka, Japan). Quantitative PCR was performed with SYBR Green Master Mix (Toyobo) on a Roche LightCycler 480 II system (Roche, Basel, Switzerland). *GAPDH* was used as the endogenous control. The primer sequences are listed in Table 2.

**Table 2:** qPCR primer sequences

Gene	Species	Sequence
*FTO*	Mouse	TTCTGTCTGCCATCCTGGTC TCGGAAACCACGTCTGTGAG
*PACS2*	Mouse	TAGCCTGACCCTGAAGAAGCTG CACTTGTCCACTAGGAGGCAACAC
*YTHDF1*	Mouse	CGTGGACCCCCAGAGAACAAA AACTGGACAGGTAAGGATCACTC
*YTHDF2*	Mouse	GAGCAGAGACCAAAAGGTCAAG CTGTGGGCTCAAGTAAGGTTC
*YTHDC2*	Mouse	CTGTTACTGTCCTGGTGTTCTGT CATCTCACTGTCACTGCTGTCA
*EIF3A*	Mouse	TATGGTCAGGTTCAGTGTGCTA AGGCGGAGTATGGTATTGTTCT
*IGF2BP1*	Mouse	CAAATGGGTGACTATGGACTGAC TGAGGACTGAGGTAGTTCATCC
*GAPDH*	Mouse	GCATCTTCTTGTGCAGTGCC TACGGCCAAATCCGTTCACA

### Western blotting

Total protein was extracted from cultured cells by using lysis buffer (KeyGen Biotech, Nanjing, China) supplemented with protease and phosphatase inhibitors (Selleck Chemicals, Houston, USA). The protein concentration was determined with a BCA assay kit (Beyotime Biotechnology, Shanghai, China). Proteins were denatured in 5× SDS loading buffer (Biosharp, Hefei, China) by boiling for 10 min, after which they were separated via 10% SDS‒PAGE and transferred to polyvinylidene fluoride (PVDF) membranes (Merck Millipore, Darmstadt, Germany). After being blocked with 5% nonfat milk for 1.5 h at room temperature, the membranes were incubated overnight at 4 °C with specific primary antibodies. The membranes were subsequently incubated with an HRP-conjugated secondary antibody (Bioworld, Bloomington, USA) for 2 h at room temperature. The protein bands were visualized by using an enhanced chemiluminescence (ECL) kit (Biosharp). The following primary antibodies were used: FTO (27226-1-AP, Proteintech), PACS2 (30172-1-AP, Proteintech), YTHDF2 (24744-1-AP, Proteintech), PPARγ (#2435, Cell Signaling Technology, Danvers, USA), CD36 (ab252922, Abcam), PLIN2 (15294-1-AP, Proteintech), Lamin B1 (66095-1-Ig, Proteintech), and GAPDH (60004-1-Ig, Proteintech).

### Isolation of nuclear and cytoplasmic proteins

Nuclear and cytoplasmic proteins were isolated from macrophages by using a commercial extraction kit (Beyotime) according to the manufacturer’s instructions. Briefly, the cells were scraped and pelleted via centrifugation. The pellet was resuspended in cytoplasmic extraction reagent A containing PMSF, vortexed for 5 s, and incubated on ice for 15 min. Cytoplasmic extraction reagent B was subsequently added, followed by vortexing for 5 s and incubation on ice for 1 min. After centrifugation at 16,000 × g for 5 min at 4 °C, the supernatant (cytoplasmic fraction) was collected. The pellet was subsequently washed and recentrifuged to remove residual supernatant. Nuclear extraction reagent supplemented with PMSF was added to the pellet, followed by vigorous vortexing for 5 s at 10 min intervals for 30 min. The sample was subsequently centrifuged at 16,000 × g for 10 min at 4 °C to collect the nuclear fraction in the supernatant. Western blotting was performed as previously described.

### Oil Red O staining

After treatment, the macrophages on the slides were stimulated with Ox-LDL for 24 h, fixed with 4% paraformaldehyde for 15 min, and washed with PBS. The cells were then stained with 0.5% Oil Red O solution for 15 min. Images were acquired by using light microscopy, and lipid accumulation was quantified with ImageJ software.

### Dil-OxLDL uptake assay

The macrophages were incubated with 20 µg/mL fluorescent-labeled oxidized LDL (Dil-OxLDL) (Yiyuan Biotechnologies) for 6 h at 37 °C. After the cells were washed with PBS to remove noninternalized particles, cellular Dil-OxLDL uptake was visualized under a Zeiss Axio Vert.A1 fluorescence microscope and quantified by measuring the mean fluorescence intensity (MFI) using ImageJ.

### BODIPY staining of lipid droplets

To visualize lipid droplets, the macrophages were incubated with 10 µM BODIPY 493/503 (HY-W090090; MCE, New Jersey, USA) for 30 min at 37 °C in the dark. The nuclei were counterstained with DAPI. After the cells were washed with PBS, fluorescence images were captured via laser scanning confocal microscopy. Lipid droplet parameters were quantified by using ImageJ.

### m^6^A dot blot assays

Total RNA was extracted by using TRIzol reagent and normalized to a concentration of 0.4 µg/µL. After denaturation at 95 °C for 5 min and rapid cooling on ice, the RNA samples were blotted onto a nitrocellulose membrane (Biosharp). The membrane was UV cross-linked, blocked with 5% nonfat milk, and incubated overnight at 4 °C with an anti-m^6^A antibody (68055-1-Ig, 1:1000, Proteintech). The membranes were subsequently incubated with an HRP-conjugated secondary antibody for 2 h at room temperature, and signals were detected by using an ECL kit. Total RNA loading was verified via methylene blue staining.

### MeRIP-qPCR

The m^6^A enrichment on *PACS2* mRNA was assessed by using an MeRIP m^6^A kit (5203-2, BersinBio, Guangzhou, China). Briefly, RNA was fragmented and immunoprecipitated with an m^6^A-specific antibody conjugated to protein A/G magnetic beads. After being washed, the bound RNA was eluted and reverse-transcribed into cDNA. The abundance of *PACS2* mRNA in immunoprecipitated and input samples was quantified via RT-qPCR.

### RNA stability test

Cells were seeded in 12-well plates and transfected with either an FTO overexpression plasmid or a YTHDF2-targeting siRNA. Transcription was halted via the addition of actinomycin D (5 µg/ml; HY-17559, MCE). Cells were collected at 0, 3, 6, and 9 h posttreatment. Total RNA was extracted, and *PACS2* mRNA levels were measured via RT–qPCR. The values were normalized to the level at 0 h to determine the RNA half-life.

### GEO data analysis

Expression data from the GEO series GSE41571 were downloaded from the GEO database (https://www.ncbi.nlm.nih.gov/geo/). Differential expression analysis between ruptured and stable plaques was conducted by using the “limma” package in R. Genes exhibiting |log2-fold change (FC)| > 1.5 and *p* value < 0.05 (Benjamini-Hochberg correction) were defined as differentially expressed genes (DEGs).

### Weighted gene coexpression network analysis (WGCNA) and kyoto encyclopedia of genes and genomes (KEGG) pathway enrichment analysis

Coexpression networks were constructed by using the “WGCNA” R package. We used gene expression data to construct a coexpression network. First, genes with low variability (those among the bottom 50% according to the median absolute deviation, MAD) were removed. The outlier genes and samples were further filtered by using the “goodSamplesGenes” function in the WGCNA R package. We then constructed a scale-free coexpression network with WGCNA. Pearson’s correlation coefficients were calculated for all of the gene pairs, and a weighted adjacency matrix was generated by using a power function (Amn=∣Cmn∣βAmn=∣Cmn∣β), with β = 10. The adjacency matrix was converted to a topological overlap matrix (TOM) to measure gene connectivity, and dissimilarity (1 − TOM1−TOM) was calculated. Genes were grouped into modules by using hierarchical clustering based on TOM dissimilarity, with a minimum module size of 30 and a sensitivity set to 3. Modules exhibiting eigengene dissimilarity of less than 0.25 were merged. In total, 34 coexpression modules were obtained. Each module was assigned a unique color label, and module eigengenes were used to represent overall expression patterns. Key genes within candidate modules were identified by using the criteria |Module Membership| > 0.8 and |Gene Significance| > 0.2.

KEGG pathway enrichment analysis was subsequently performed on the robust gene set (derived from the intersection between key WGCNA modules and the previously identified DEGs) by using the “clusterProfiler” package. Terms with an adjusted *p* value < 0.05 were considered to be statistically significant [20].

### Protein-RNA molecular docking

The structure of the complex between the FTO protein structure (FTO_HUMAN, AlphaFold ID: AF-Q9C0B1-F1-v6) and the *PACS2* mRNA fragment (5′-UAGCCUGACCCUGAAGAAGC-3′) was predicted by using AlphaFold3. Protein-RNA interaction analysis was conducted with HDOCKlite v1.1. A comprehensive description and systematic analysis of the binding interface of protein-nucleic acid complexes were performed by using the PLIP interaction analysis platform. The binding free energy (δ^i^G) was calculated by using PDBePISA [21]. Additionally, interaction-related details were supplemented by using PyMOL (https://www.pymol.org/) [22].

### Protein-protein molecular docking

Crystal structures of PACS2 were obtained from the AlphaFold Protein Structure Database (Q86VP3), and those of PPARγ were obtained from the PDB (3DZY: D). The PDB files of these proteins were subsequently imported into HDOCK (http://hdock.phys.hust.edu.cn/) [22] and HawkDock (https://cadd.zju.edu.cn/hawkdock/) [23]. A comprehensive description and systematic analysis of the binding interface of protein-protein complexes were performed by using the PLIP interaction analysis platform. Additionally, interaction-related details were supplemented by using PyMOL (https://www.pymol.org/) [24].

### Immunoprecipitation

To investigate the protein-protein interaction between PACS2 and PPARγ, coimmunoprecipitation (Co-IP) was performed by using a commercial immunoprecipitation kit (Beyotime) following the manufacturer’s protocol. Cells were lysed in IP-compatible lysis buffer containing protease inhibitors. The lysates were incubated with protein A/G magnetic beads preconjugated with an anti-PACS2 antibody. After being washed, the immunoprecipitated complexes were eluted and analyzed via Western blotting using antibodies against PACS2 and PPARγ.

### Statistical analysis

The data are presented as the mean ± standard deviation (SD). Statistical analyses were conducted by using GraphPad Prism software (GraphPad, San Diego, CA, USA). Comparisons between the groups were conducted by using the Student’s t test or one-way analysis of variance (ANOVA) followed by the Tukey’s post hoc test. *P* values < 0.05 were considered to indicate statistical significance.

## Results

### FTO is downregulated in macrophages upon Ox-LDL stimulation

**Fig. 1:**
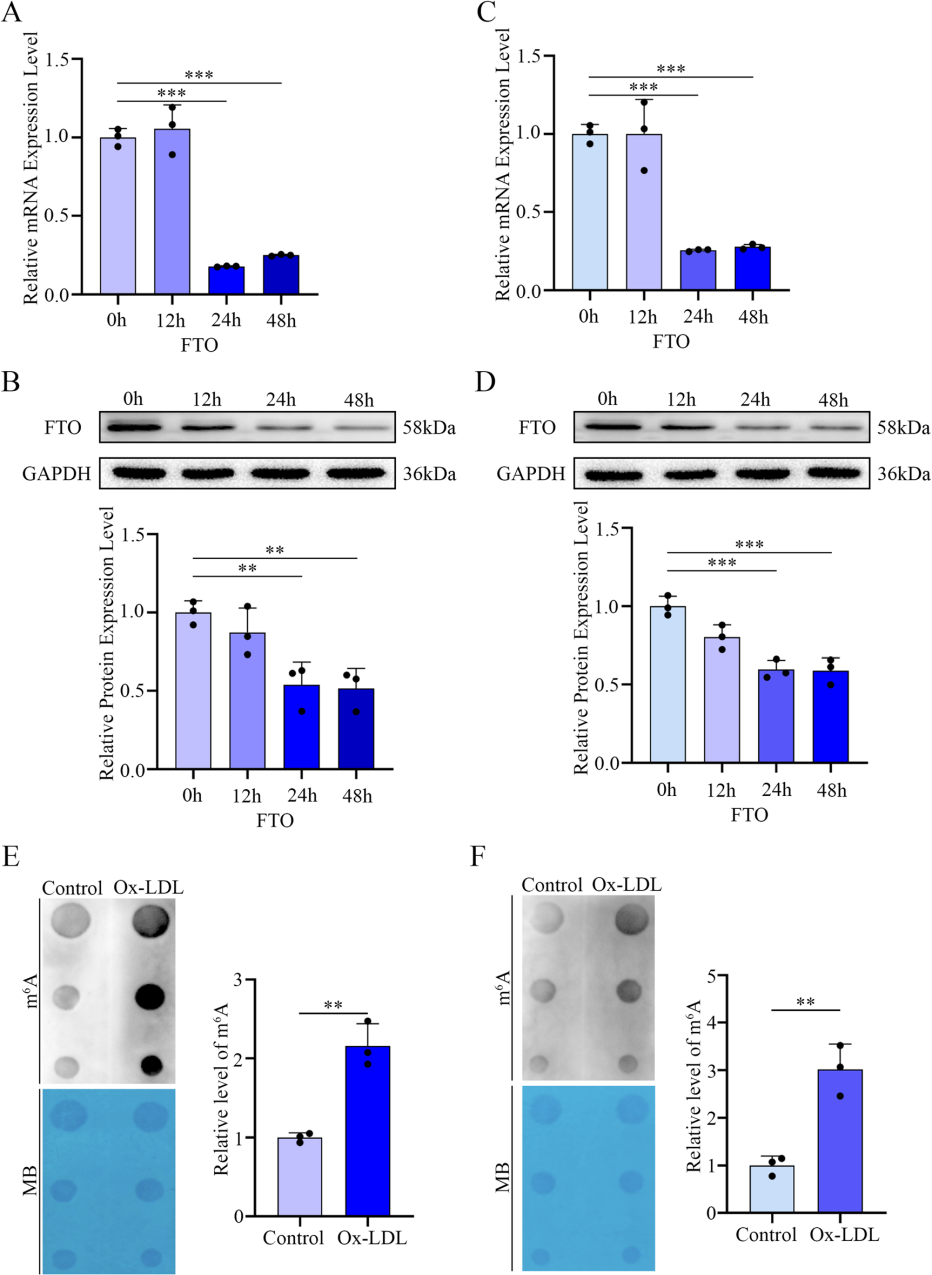
FTO in macrophages is downregulated under conditions of Ox-LDL stimulation. (**A**) *FTO* mRNA levels in PMs treated with 100 µg/mL Ox-LDL for various durations (*n* = 3). (**B**) Representative Western blotting images and quantification of FTO levels in PMs treated with 100 µg/mL Ox-LDL for different durations (*n* = 3). (**C**) *FTO* mRNA levels in RAW264.7 cells treated with 100 µg/mL Ox-LDL for different durations (*n* = 3). (**D**) Representative Western blotting images and quantification of FTO levels in RAW264.7 cells treated with 100 µg/mL Ox-LDL for different durations (*n* = 3). (**E**) Dot blot assay using an anti-m^6^A antibody in 100 µg/mL Ox-LDL-stimulated PMs. Methylene blue staining was included as a loading control. (**F**) Dot blot assay using an anti-m^6^A antibody in 100 µg/mL Ox-LDL-stimulated RAW264.7 cells. Methylene blue staining was included as a loading control

Given the critical role of Ox-LDL in atherosclerosis, we treated PMs and RAW264.7 cells with 100 µg/mL Ox-LDL. Time-course analysis revealed a progressive decrease in FTO expression at both the mRNA and protein levels (Fig. 1A-D). Consistent with these findings, dot blot assays revealed a significant increase in global m^6^A modification in Ox-LDL-stimulated macrophages compared with controls (Fig. 1E and F). These results suggest that FTO may play important roles in macrophage biology and atherogenesis.

### FTO attenuates foam cell formation by reducing lipid uptake and deposition in macrophages

**Fig. 2:**
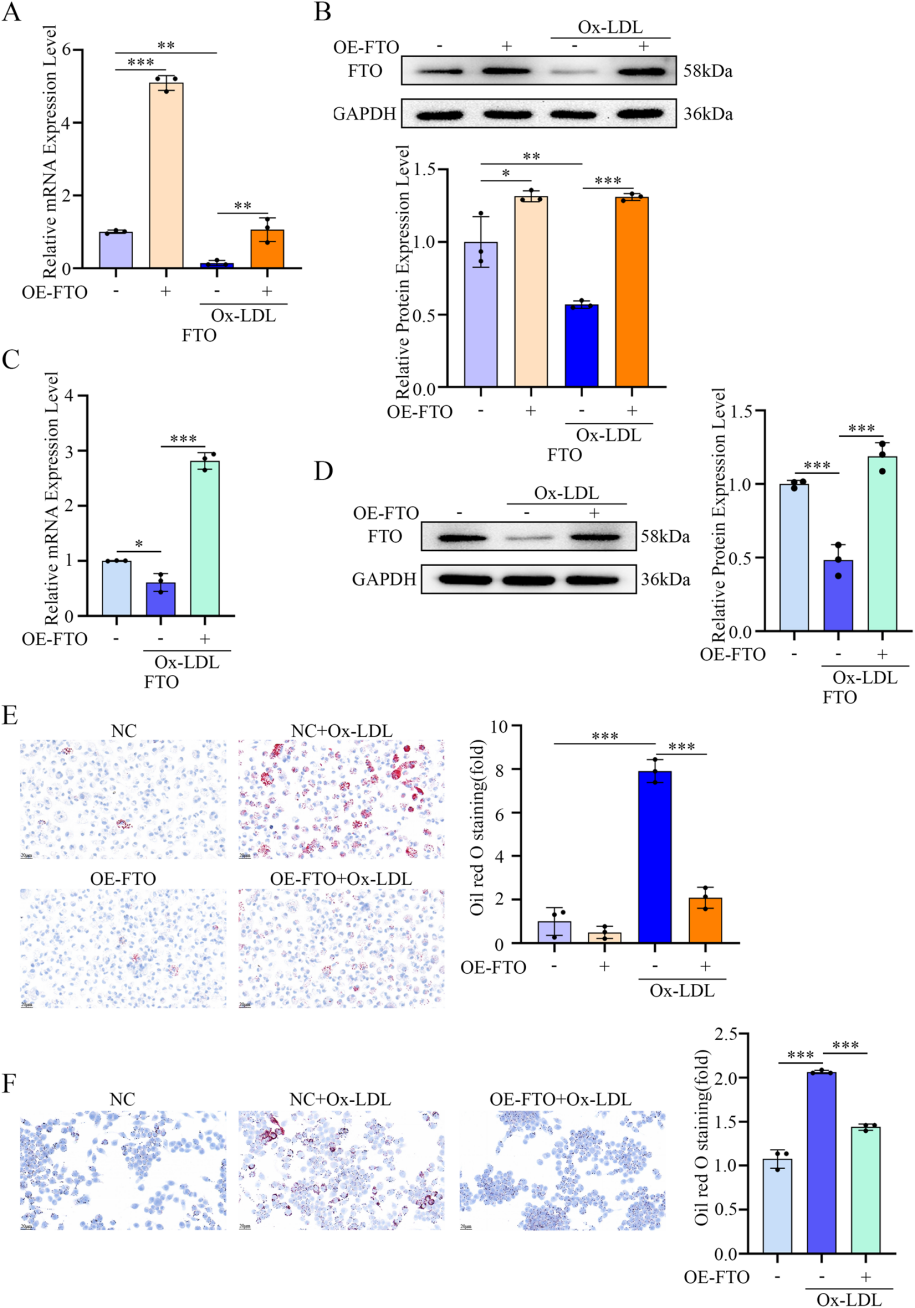
FTO mediates Ox-LDL-induced foam cell formation in macrophages. (**A**) RT-qPCR analysis of *FTO* mRNA levels in PMs pretreated with OE-NC or OE-*FTO* and stimulated with Ox-LDL (*n* = 3). (**B**) Representative Western blotting images and quantification of FTO levels in PMs pretreated with OE-NC or OE-*FTO* and stimulated with Ox-LDL (*n* = 3). (**C**) RT-qPCR analysis of *FTO* mRNA levels in RAW264.7 cells pretreated with OE-NC or OE-*FTO* and stimulated with Ox-LDL (*n* = 3). (**D**) Representative Western blotting images and quantification of FTO levels in RAW264.7 cells pretreated with OE-NC or OE-*FTO* and stimulated with Ox-LDL (*n* = 3). (**E**) Representative Oil Red O images showing foam formation in PMs pretreated with OE-NC or OE-*FTO* and stimulated with Ox-LDL (*n* = 3). (**F**) Representative Oil Red O images showing foam formation in RAW264.7 cells pretreated with OE-NC or OE-*FTO* and stimulated with Ox-LDL (*n* = 3)

As macrophage-derived foam cells are a hallmark of atherosclerosis across all stages [25], we hypothesized that FTO may regulate lipid homeostasis in macrophages. To test whether FTO influences foam cell formation, we overexpressed FTO in macrophages prior to Ox-LDL stimulation. As shown in Fig. 2A-D, Ox-LDL treatment reduced FTO expression at both the mRNA and protein levels in PMs and RAW264.7 cells, whereas FTO overexpression effectively restored its expression. Importantly, FTO overexpression markedly attenuated Ox-LDL-induced foam cell formation (Fig. 2E and F).

**Fig. 3:**
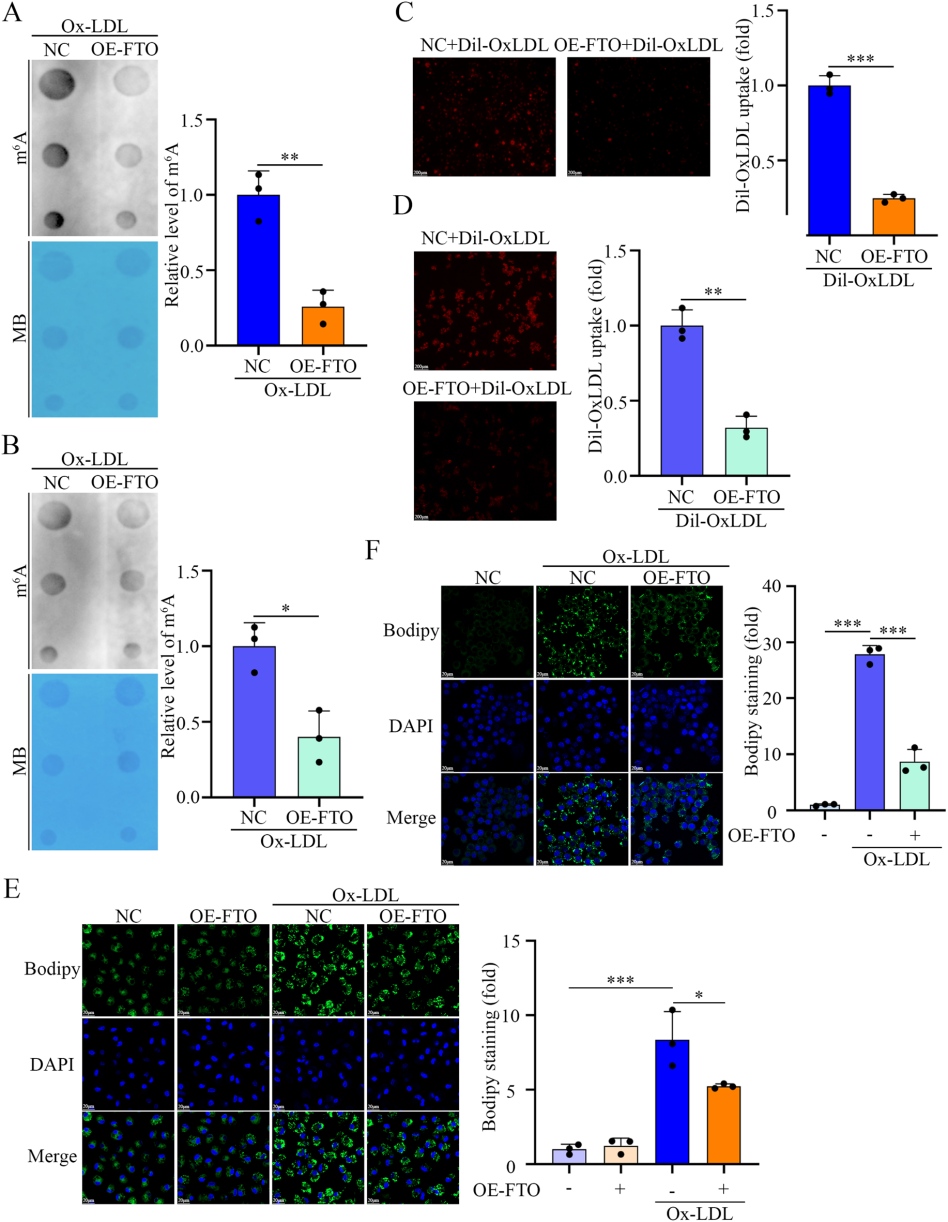
FTO alleviates Ox-LDL-induced lipid uptake and deposition in macrophages. (**A**) Dot blot assay using an anti-m^6^A antibody in PMs pretreated with OE-NC or OE-*FTO* and stimulated with Ox-LDL. Methylene blue staining was included as a loading control (*n* = 3). (**B**) Dot blot assay using an anti-m^6^A antibody in RAW264.7 cells pretreated with OE-NC or OE-*FTO* and stimulated with Ox-LDL. Methylene blue staining was included as a loading control (*n* = 3). (**C**) Representative fluorescence images illustrating lipid uptake and quantitative data on fluorescence intensity in PMs pretreated with OE-NC or OE-*FTO* and stimulated with Dil-OxLDL. Scale bars = 200 μm (*n* = 3). (**D**) Representative fluorescence images illustrating lipid uptake and quantitative data on fluorescence intensity in RAW264.7 cells pretreated with OE-NC or OE-*FTO* and stimulated with Dil-OxLDL. Scale bars = 200 μm (*n* = 3). (**E**) Representative fluorescence images illustrating lipid deposition and quantitative data on fluorescence intensity in PMs pretreated with OE-NC or OE-*FTO* and stimulated with OxLDL. Scale bars = 20 μm (*n* = 3). (**F**) Representative fluorescence images illustrating lipid deposition and quantitative data on fluorescence intensity in RAW264.7 cells pretreated with OE-NC or OE-*FTO* and stimulated with OxLDL. Scale bars = 20 μm (*n* = 3)

Furthermore, global m^6^A levels were significantly lower in FTO-overexpressing macrophages than in NC macrophages (even after Ox-LDL stimulation), thus indicating that FTO critically regulates m^6^A modification in macrophages (Fig. 3A and B). We next assessed lipid uptake by using Dil-OxLDL and observed a pronounced reduction in lipid internalization upon FTO overexpression (Fig. 3C and D). Additionally, BODIPY 493/503 staining revealed that lipid droplet accumulation induced by Ox-LDL was significantly suppressed in FTO-overexpressing macrophages (Fig. 3E and F), thus suggesting that FTO inhibits lipid deposition.

Collectively, these findings demonstrate that FTO overexpression mitigates foam cell formation by decreasing both lipid uptake and deposition, which are likely achieved via the modulation of m^6^A-dependent pathways.

### Macrophage-specific FTO overexpression attenuates atherosclerosis progression in vivo

**Fig. 4:**
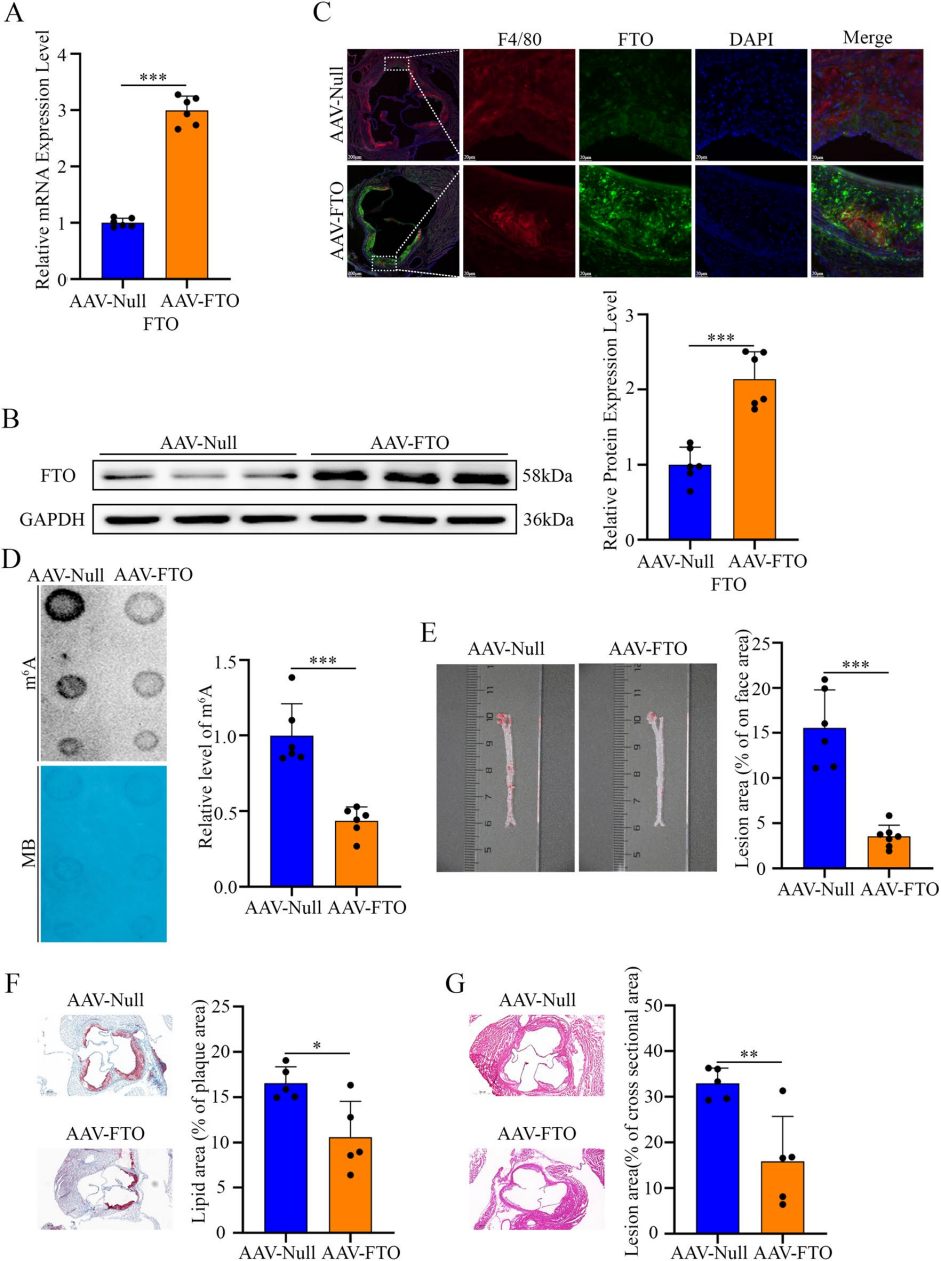
Macrophage-specific FTO overexpression attenuates atherosclerotic plaque formation. (**A**) mRNA levels of FTO in the PMs of *ApoE*^*−/−*^ mice were measured after intravenous injection of AAV-Null or AAV-FTO through the tail vein (*n* = 6). (**B**) Representative Western blotting images of FTO levels in the PMs of *ApoE*^*−/−*^ mice treated with AAV-Null or AAV-FTO (*n* = 6). (**C**) Representative fluorescence images illustrating FTO expression in macrophages in frozen aortic root sections from *ApoE*^*−/−*^ mice treated with AAV-Null or AAV-FTO. (**D**) Dot blot assay using an anti-m^6^A antibody in aortic plaques pretreated with AAV-Null or AAV-FTO. Methylene blue staining was included as a loading control (*n* = 6). (**E**) Representative Oil Red O staining images and quantification of atherosclerotic lesions in whole aortas from *ApoE*^*−/−*^ mice treated with AAV-Null or AAV-FTO (*n* = 6). (**F**) Representative Oil Red O staining images and quantification of atherosclerotic lesions in the aortic roots of *ApoE*^*−/−*^ mice treated with AAV-Null or AAV-FTO (*n* = 5). (**G**) Representative H&E staining images and quantification of the atherosclerotic lesion area in the aortic roots of *ApoE*^*−/−*^ mice treated with AAV-Null or AAV-FTO (*n* = 5)

To investigate the role of macrophage FTO in atherosclerosis, *ApoE*^*−/−*^ mice were injected with an adeno-associated virus enabling macrophage-specific FTO overexpression (AAV-FTO). RT-qPCR and Western blotting confirmed that FTO expression was elevated in macrophages from AAV-FTO mice compared with control mice (Fig. 4A and B). Immunofluorescence staining of plaque sections further verified the increase in FTO expression in macrophages (Fig. 4C). Moreover, reduced global m^6^A levels were detected in aortic arch plaque tissues from AAV-FTO mice (Fig. 4D). En face analysis of the aortas revealed a significant reduction in the atherosclerotic lesion area in AAV-FTO mice (Fig. 4E). Consistent with this observation, histological examination revealed that macrophage-specific FTO overexpression markedly decreased the plaque burden in the aortic root, as demonstrated by H&E and Oil Red O staining (Fig. 4F and G). We also measured changes in the body weight and blood lipid levels of the mice. The results showed that overexpression of FTO did not affect the body weight and blood lipid levels of the mice (Supplementary Fig. 1A, B).

Collectively, these in vivo findings demonstrate that targeted FTO overexpression in macrophages effectively attenuates the development and progression of atherosclerotic plaques.

### FTO modulates macrophage lipid uptake and deposition via PACS2 regulation

**Fig. 5:**
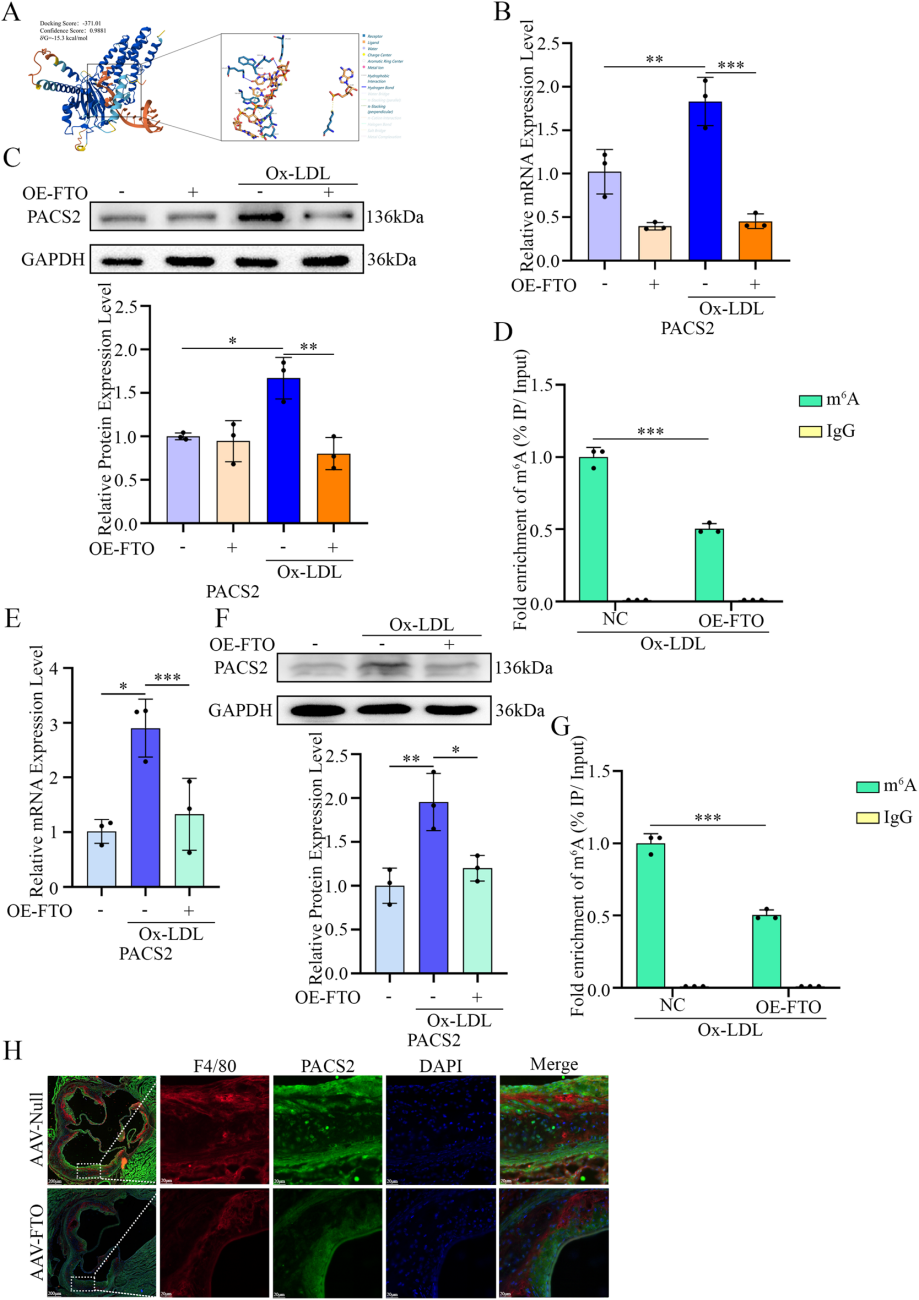
Overexpression of FTO reduced the level of m^6^A modification of PACS2. (**A**) The predicted interaction mode between FTO and PACS2. (**B**) RT-qPCR analysis of *PACS2* mRNA levels in PMs pretreated with OE-NC or OE-*FTO* and stimulated with Ox-LDL (*n* = 3). (**C**) Representative Western blotting images and quantification of PACS2 levels in PMs pretreated with OE-NC or OE-*FTO* and stimulated with Ox-LDL (*n* = 3). (**D**) MeRIP-qPCR assays revealed that the overexpression of FTO significantly reduced the level of PACS2 m^6^A modification in PMs. (**E**) RT-qPCR analysis of *PACS2* mRNA levels in RAW264.7 cells pretreated with OE-NC or OE-*FTO* and stimulated with Ox-LDL (*n* = 3). (**F**) Representative Western blotting images and quantification of PACS2 levels in RAW264.7 cells pretreated with OE-NC or OE-*FTO* and stimulated with Ox-LDL (*n* = 3). (**G**) MeRIP-qPCR assays revealed that the overexpression of FTO significantly reduced the level of PACS2 m^6^A modification in RAW264.7 cells. (**H**) Representative immunofluorescence images of PACS2 protein levels in macrophages in frozen aortic root sections from *ApoE*^*−/−*^ mice treated with AAV-Null or AAV-FTO

Our previous study revealed that PACS2 promotes foam cell formation by enhancing lipid uptake in macrophages [18]. Given that FTO acts as an m^6^A demethylase and serves as an RNA modification “eraser”, we hypothesized that FTO may regulate macrophage foaming by modulating m^6^A methylation of PACS2. Molecular docking analysis using AutoDock software [26] revealed a direct interaction between FTO and PACS2 (Fig. 5A).

In vitro, Ox-LDL stimulation increased both the mRNA and protein expression of PACS2, whereas FTO overexpression significantly suppressed this upregulation (Fig. 5B, C, E and F). MeRIP-qPCR analysis revealed that FTO overexpression reduced m^6^A enrichment on PACS2 transcripts (Fig. 5D and G). Concordantly, decreased PACS2 expression was observed in plaque macrophages from AAV-FTO mice (Fig. 5H).

**Fig. 6:**
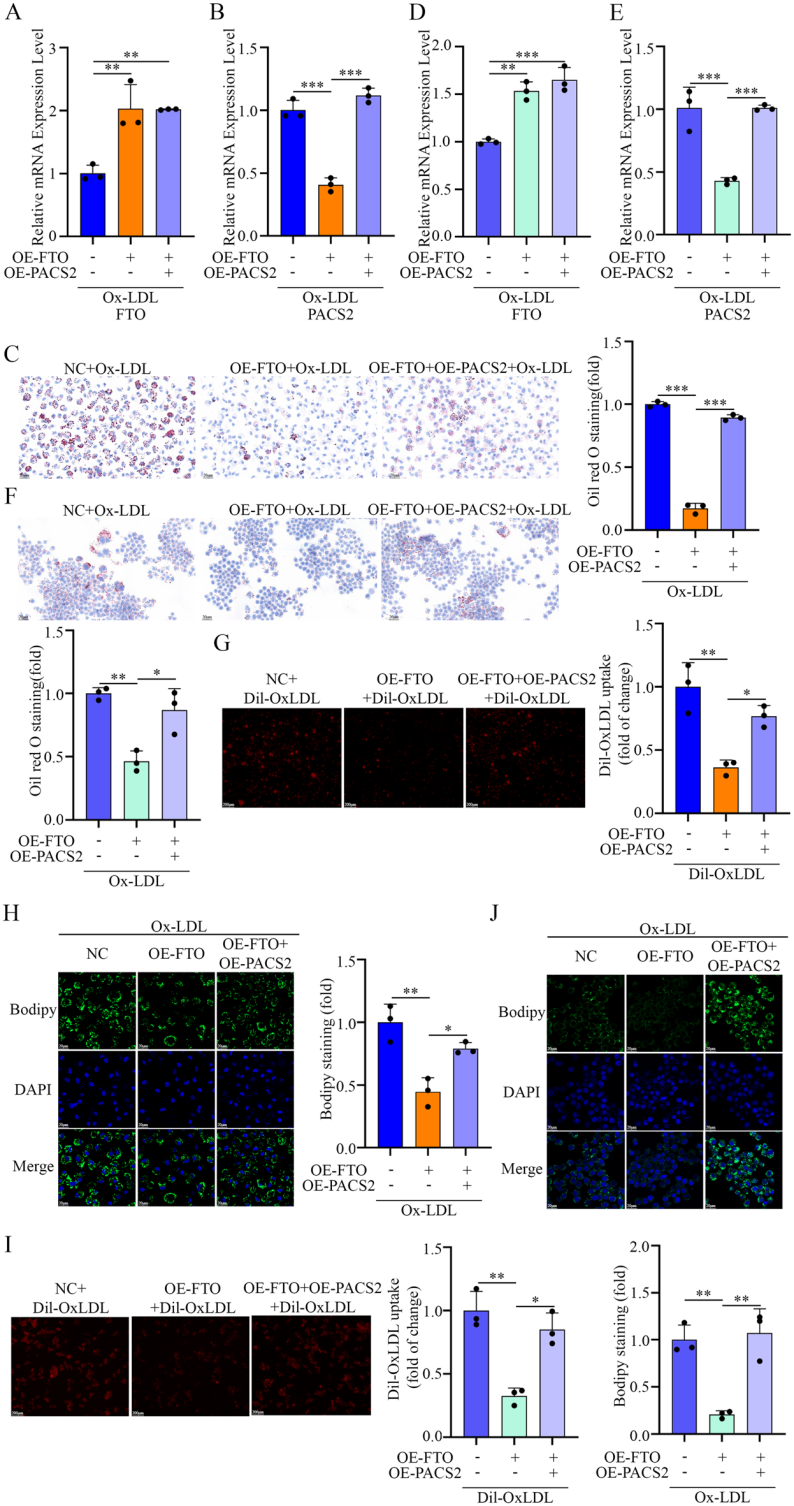
FTO affects Ox-LDL-induced lipid uptake and deposition by regulating PACS2 protein expression. (**A**) RT-qPCR analysis of *FTO* mRNA levels in PMs pretreated with OE-*FTO* or OE-*PACS2* and stimulated with Ox-LDL (*n* = 3). (**B**) RT-qPCR analysis of *PACS2* mRNA levels in PMs pretreated with OE-*FTO* or OE-*PACS2* and stimulated with Ox-LDL (*n* = 3). (**C**) Representative Oil Red O images showing foam formation in PMs pretreated with OE-*FTO* or OE-*PACS2* and stimulated with Ox-LDL (*n* = 3). (**D**) RT-qPCR analysis of *FTO* mRNA levels in RAW264.7 cells pretreated with OE-*FTO* or OE-*PACS2* and stimulated with Ox-LDL (*n* = 3). (**E**) RT-qPCR analysis of *PACS2* mRNA levels in RAW264.7 cells pretreated with OE-*FTO* or OE-*PACS2* and stimulated with Ox-LDL (*n* = 3). (**F**) Representative Oil Red O images showing foam formation in RAW264.7 cells pretreated with OE-*FTO* or OE-*PACS2* and stimulated with Ox-LDL (*n* = 3). (**G**) Representative fluorescence images illustrating lipid uptake and quantitative data on fluorescence intensity in PMs pretreated with OE-*FTO* or OE-*PACS2* and stimulated with Ox-LDL. Scale bars = 200 μm (*n* = 3). (**H**) Representative fluorescence images illustrating lipid deposition and quantitative data on fluorescence intensity in PMs pretreated with OE-*FTO* or OE-*PACS2* and stimulated with Ox-LDL. Scale bars = 200 μm (*n* = 3). (**I**) Representative fluorescence images illustrating lipid uptake and quantitative data on fluorescence intensity in PMs pretreated with OE-*FTO* or OE-*PACS2* and stimulated with Ox-LDL. Scale bars = 200 μm (*n* = 3). (**J**) Representative fluorescence images illustrating lipid deposition and quantitative data on fluorescence intensity in PMs pretreated with OE-*FTO* or OE-*PACS2* and stimulated with Ox-LDL. Scale bars = 200 μm (*n* = 3)

To determine whether the anti-foaming effect of FTO depends on PACS2 downregulation, we cotransfected macrophages (both PMs and RAW264.7 cells) with FTO- and PACS2-overexpressing plasmids (Fig. 6A, B, D and E). Notably, PACS2 overexpression abolished the protective effect of FTO on Ox-LDL-induced foam cell formation (Fig. 6C and F). Furthermore, Dil-OxLDL and BODIPY 493/503 staining confirmed that PACS2 overexpression reversed the FTO-mediated suppression of lipid uptake (Fig. 6G and I) and lipid accumulation (Fig. 6H and J). These rescue experiments indicate that FTO mainly alleviates macrophage foaming through the downregulation of PACS2.

### FTO regulates *PACS2* mRNA stability through YTHDF2

**Fig. 7:**
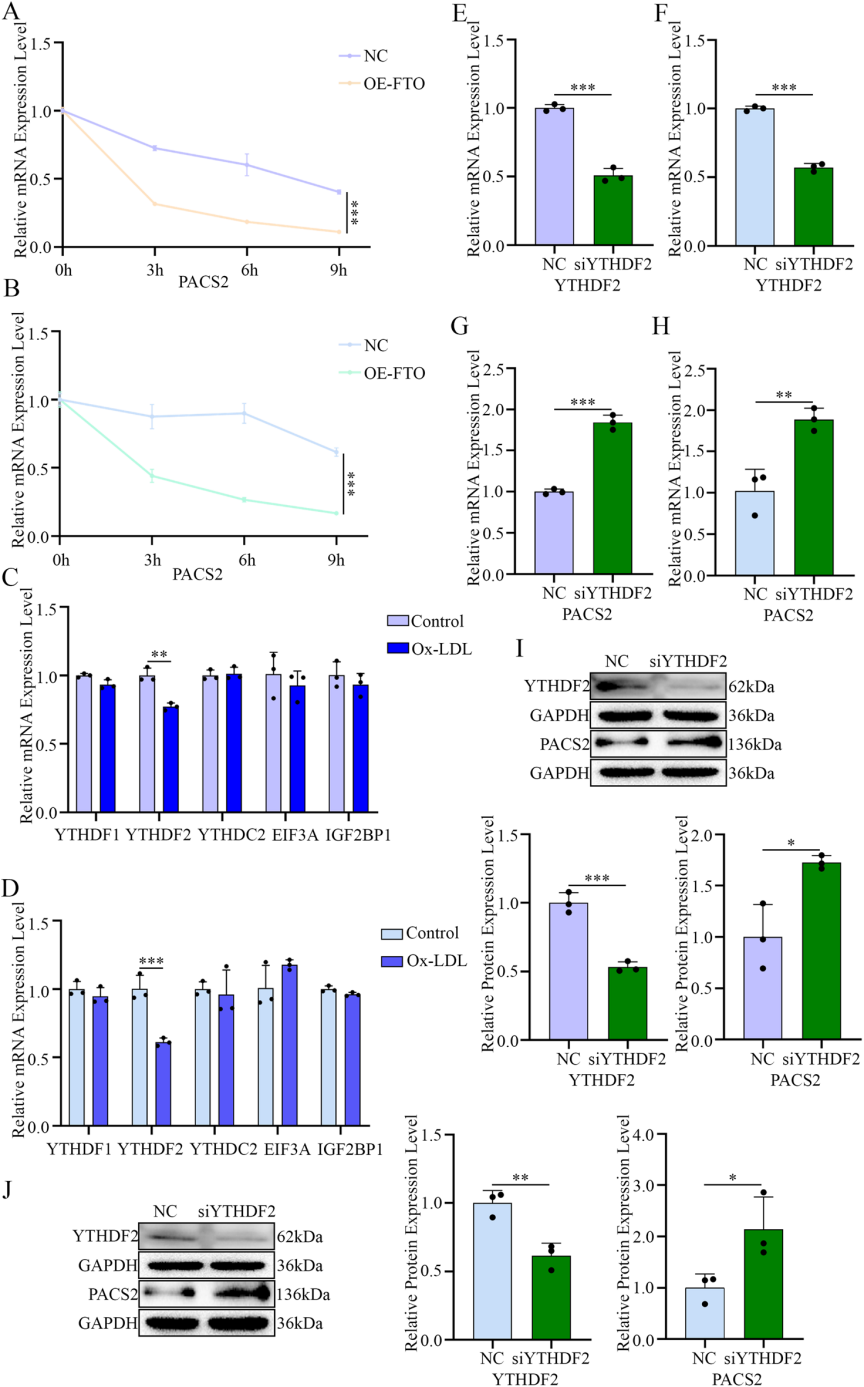
FTO inhibits the translation of *PACS2* MRNA through YTHDF2. (**A**) Effects of FTO on the half-life of *PACS2* mRNA in PMs determined via RT-qPCR. (**B**) Effects of FTO on the half-life of *PACS2* mRNA in RAW264.7 cells determined via RT-qPCR. (**C**) The expression levels of different m^6^A readers in PMs treated with 100 µg/mL Ox-LDL (*n* = 3). (**D**) The expression levels of different m^6^A readers in RAW264.7 cells treated with 100 µg/mL Ox-LDL (*n* = 3). (**E**) *YTHDF2* mRNA levels in PMs pretreated with si-*NC* or si-*YTHDF2* (*n* = 3). (**F**) *YTHDF2* mRNA levels in PMs pretreated with si-*NC* or si-*YTHDF2* (*n* = 3). (**G**) *PACS2* mRNA levels in PMs pretreated with si-*NC* or si-*YTHDF2* (*n* = 3). (**H**) *PACS2* mRNA levels in PMs pretreated with si-*NC* or si-*YTHDF2* (*n* = 3). (**I**) Representative Western blotting images and quantification of YTHDF2 and PACS2 levels in PMs pretreated with si-*NC* or si-*YTHDF2* (*n* = 3). (**J**) Representative Western blotting images and quantification of YTHDF2 and PACS2 levels in RAW264.7 cells pretreated with si-*NC* or si-*YTHDF2* (*n* = 3)

Given the well-established role of m^6^A modification in modulating mRNA stability and decay [27], we assessed the half-life of *PACS2* mRNA following transcriptional inhibition with actinomycin D. The overexpression of FTO significantly shortened the half-life of *PACS2* mRNA (Fig. 7A and B), thus suggesting that FTO-mediated m^6^A demethylation reduces PACS2 transcript stability and promotes its decay, thereby attenuating macrophage foam cell formation.

**Fig. 8:**
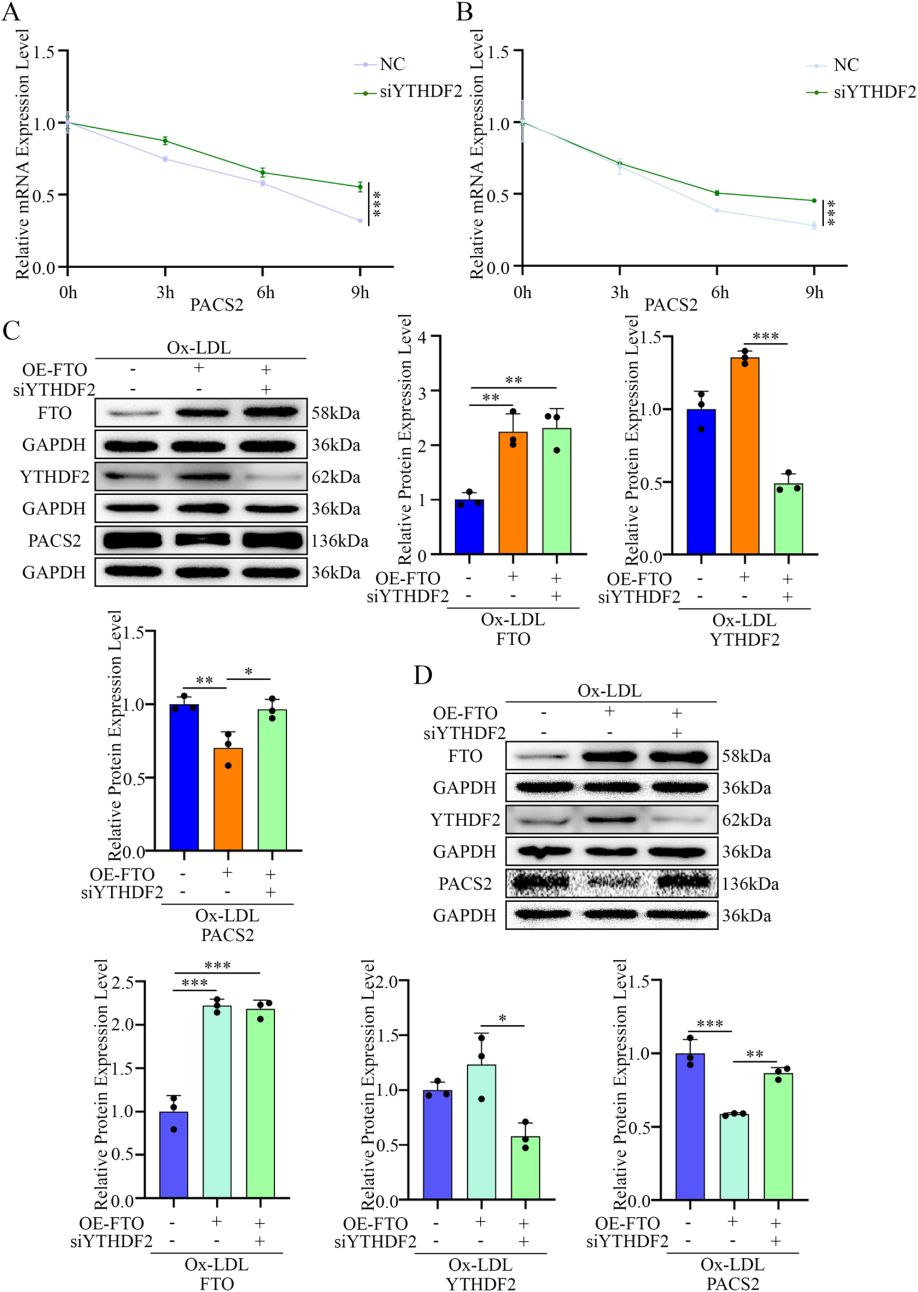
FTO inhibits PACS2 expression through YTHDF2. (**A**) Effects of YTHDF2 on the half-life of *PACS2* mRNA in PMs pretreated with si-*NC* or si-*YTHDF2* (*n* = 3). (**B**) Effects of YTHDF2 on the half-life of *PACS2* mRNA in RAW264.7 cells pretreated with si-*NC* or si-*YTHDF2* (*n* = 3). (**C**) The expression of FTO, YTHDF2, and PACS2 in PMs pretreated with OE-*FTO* or si-*YTHDF2* and stimulated with Ox-LDL was analyzed via Western blotting (*n* = 3). (**D**) The expression of FTO, YTHDF2, and PACS2 in RAW264.7 cells pretreated with OE-*FTO* or si-*YTHDF2* and stimulated with Ox-LDL was analyzed via Western blotting (*n* = 3)

We subsequently screened major m^6^A readers (including YTHDF1, YTHDF2, YTHDC2, EIF3A, and IGF2BP1 [28]) and observed that the expression of YTHDF2 (which is known to bind numerous transcripts and extend their half-life when depleted [29]) was significantly downregulated in macrophages upon Ox-LDL stimulation (Fig. 7C and D). No consistent changes were observed in the other readers. The knockdown of YTHDF2 using siRNA (Fig. 7E and F) markedly increased both *PACS2* mRNA (Fig. 7G and H) and protein levels (Fig. 7I and J). Accordingly, RNA decay assays revealed that YTHDF2 silencing delayed *PACS2* mRNA degradation and enhanced its stability (Fig. 8A and B). Importantly, under Ox-LDL stimulation, YTHDF2 knockdown abolished the suppressive effect of FTO on PACS2 expression (Fig. 8C and D).

Together, these findings demonstrate that FTO downregulates PACS2 expression by promoting YTHDF2-dependent mRNA degradation.

### FTO suppresses the PPARγ-CD36/PLIN2 pathway via PACS2 downregulation

**Fig. 9:**
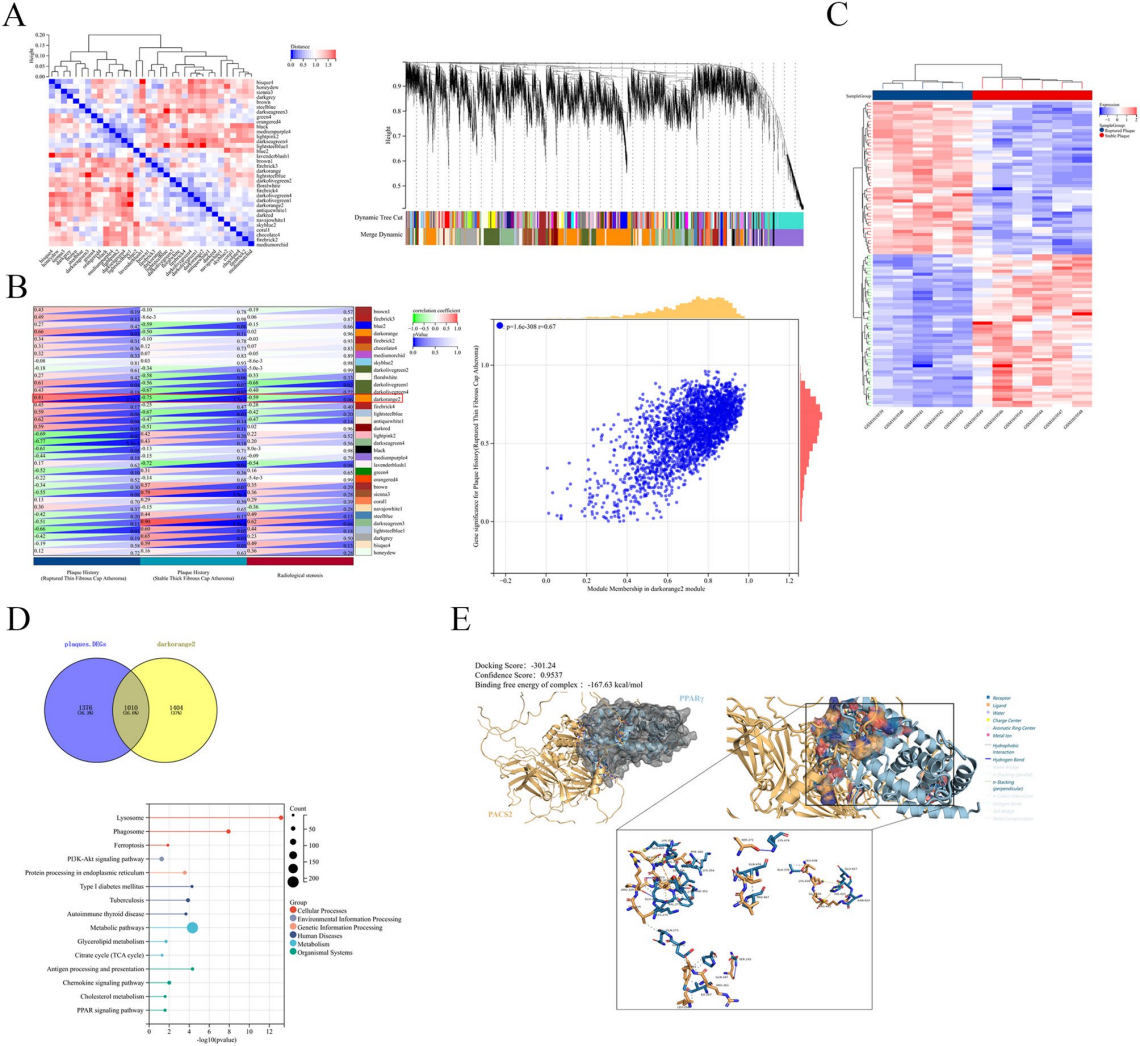
PPARγ is involved in a signaling pathway that may mediate plaque instability. (**A**–**B**) WGCNA revealed a darkorange2 module that was positively correlated with plaque rupture and negatively correlated with stability. (**C**) The heatmap displays the DEGs between unstable and stable plaques. (**D**) KEGG analysis was performed on the intersection of genes between the darkorange2 module and DEGs. (**E**) The molecular docking configuration of the PACS2-PPARγ interaction

Given the central role of macrophage foaming in plaque instability, we investigated the mechanism through which FTO regulates PACS2 to modulate lipid metabolism. WGCNA revealed a module (darkorange2) demonstrating the strongest positive correlation with plaque rupture and a negative correlation with stability (Fig. 9A and B). Analysis of a public dataset (GSE41571) containing laser-microdissected macrophages from ruptured and stable human carotid plaques [30] revealed 2386 DEGs between unstable and stable plaques, including 1490 upregulated genes and 896 downregulated genes (Fig. 9C). The intersection of the darkorange2 module with the DEGs yielded 1010 candidate genes. KEGG enrichment analysis highlighted the PPAR signaling pathway as a significantly enriched pathway (Fig. 9D), suggesting its potential involvement in atherosclerosis. Given prior evidence that PPARγ upregulation promotes plaque vulnerability [31] together with our previous finding that PACS2 increases lipid uptake via activation of the PPARγ–CD36 axis [18] and reports that PPARγ–PLIN2 signaling facilitates lipid deposition [32], we hypothesized that FTO downregulation increases PPARγ transcriptional activity through PACS2, thereby promoting CD36-mediated lipid uptake and PLIN2-mediated lipid storage. Although metabolic pathways ranked highly in the enrichment analysis, we prioritized PPAR signaling for mechanistic investigation because of its direct regulatory role in macrophage lipid handling and its established relevance to plaque progression. Molecular docking results supported a high-affinity interaction between PACS2 and the transcription factor PPARγ (Fig. 9E).

**Fig. 10:**
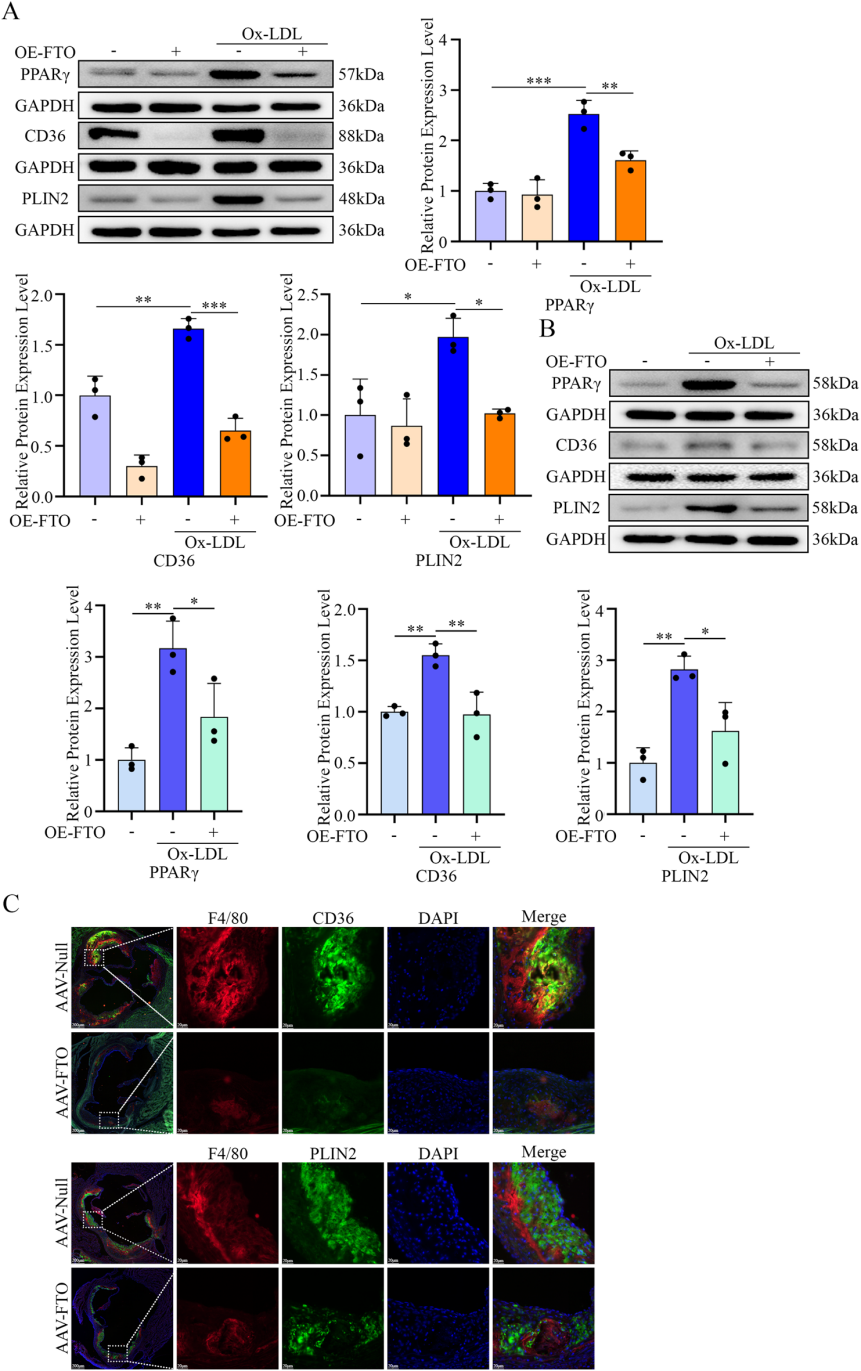
FTO regulates the lipid uptake and deposition signaling pathway. (**A**) The expression of PPARγ, CD36, and PLIN2 in PMs pretreated with OE-NC or OE-*FTO* and stimulated with Ox-LDL was analyzed via Western blotting (*n* = 3). (**B**) The expression of PPARγ, CD36, and PLIN2 in RAW264.7 cells pretreated with OE-NC or OE-*FTO* and stimulated with Ox-LDL was analyzed via Western blotting (*n* = 3). (**C**) Representative immunofluorescence images of CD36 and PLIN2 levels in macrophages in frozen aortic root sections from *ApoE*^*−/−*^ mice treated with AAV-Null or AAV-FTO

**Fig. 11:**
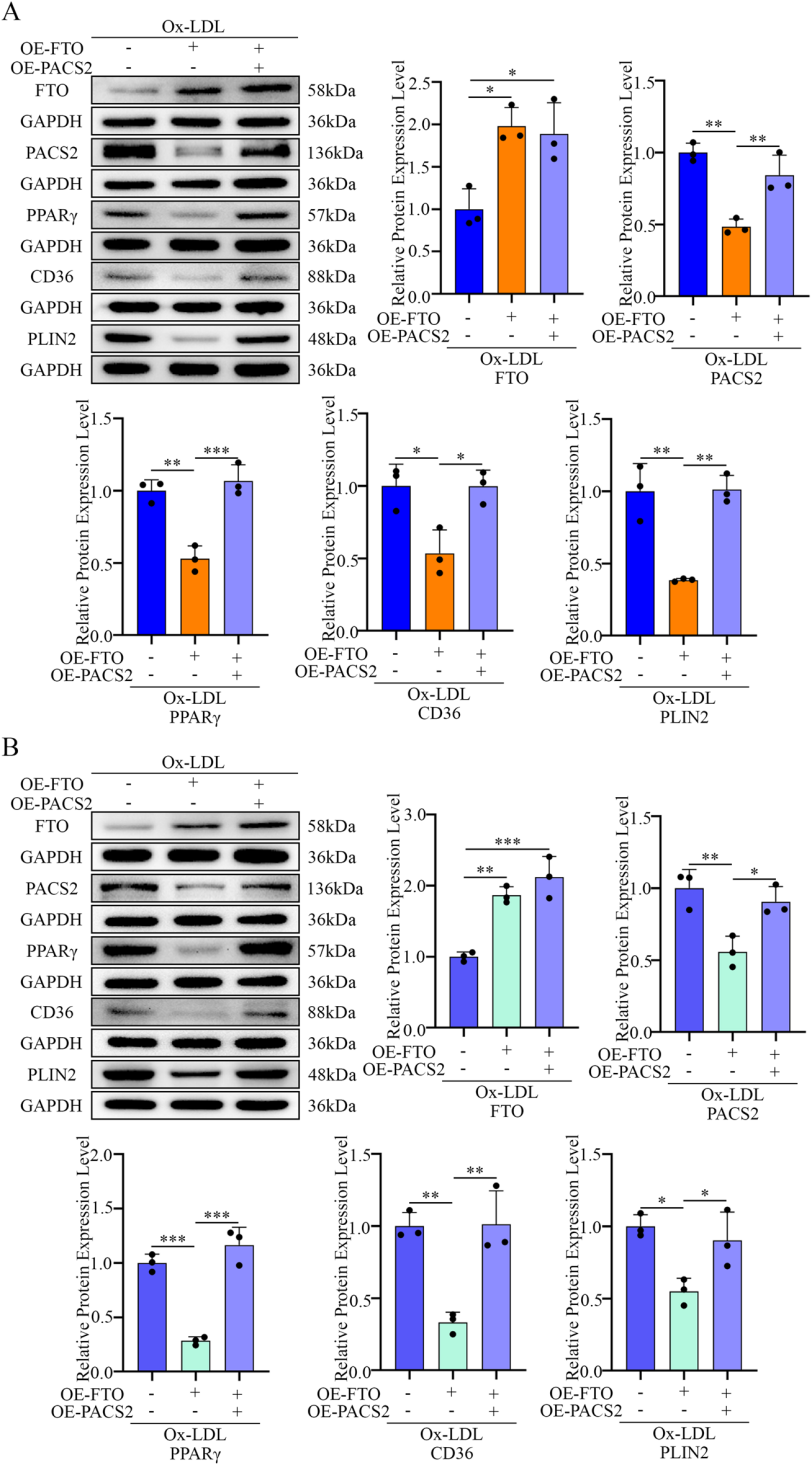
FTO regulates the lipid uptake and deposition signaling pathway via PACS2. (**A**) The expression of FTO, PACS2, PPARγ, CD36, and PLIN2 in PMs pretreated with OE-*FTO* or OE-*PACS2* and stimulated with Ox-LDL was analyzed via Western blotting (*n* = 3). (**B**) The expression of FTO, PACS2, PPARγ, CD36, and PLIN2 in RAW264.7 cells pretreated with OE-*FTO* or OE-*PACS2* and stimulated with Ox-LDL was analyzed via Western blotting (*n* = 3)

We next examined whether FTO influences components of the PPARγ pathway. The overexpression of FTO in macrophages attenuated the Ox-LDL-induced upregulation of PPARγ, CD36, and PLIN2 at the protein level in both PMs and RAW264.7 cells (Fig. 10A and B). Consistent with these findings, in vivo macrophage-specific FTO overexpression reduced CD36 and PLIN2 expression in plaque macrophages (Fig. 10C). Critically, the co-overexpression of PACS2 abrogated the suppressive effects of FTO on PPARγ, CD36, and PLIN2 expression (Fig. 11A and B).

**Fig. 12:**
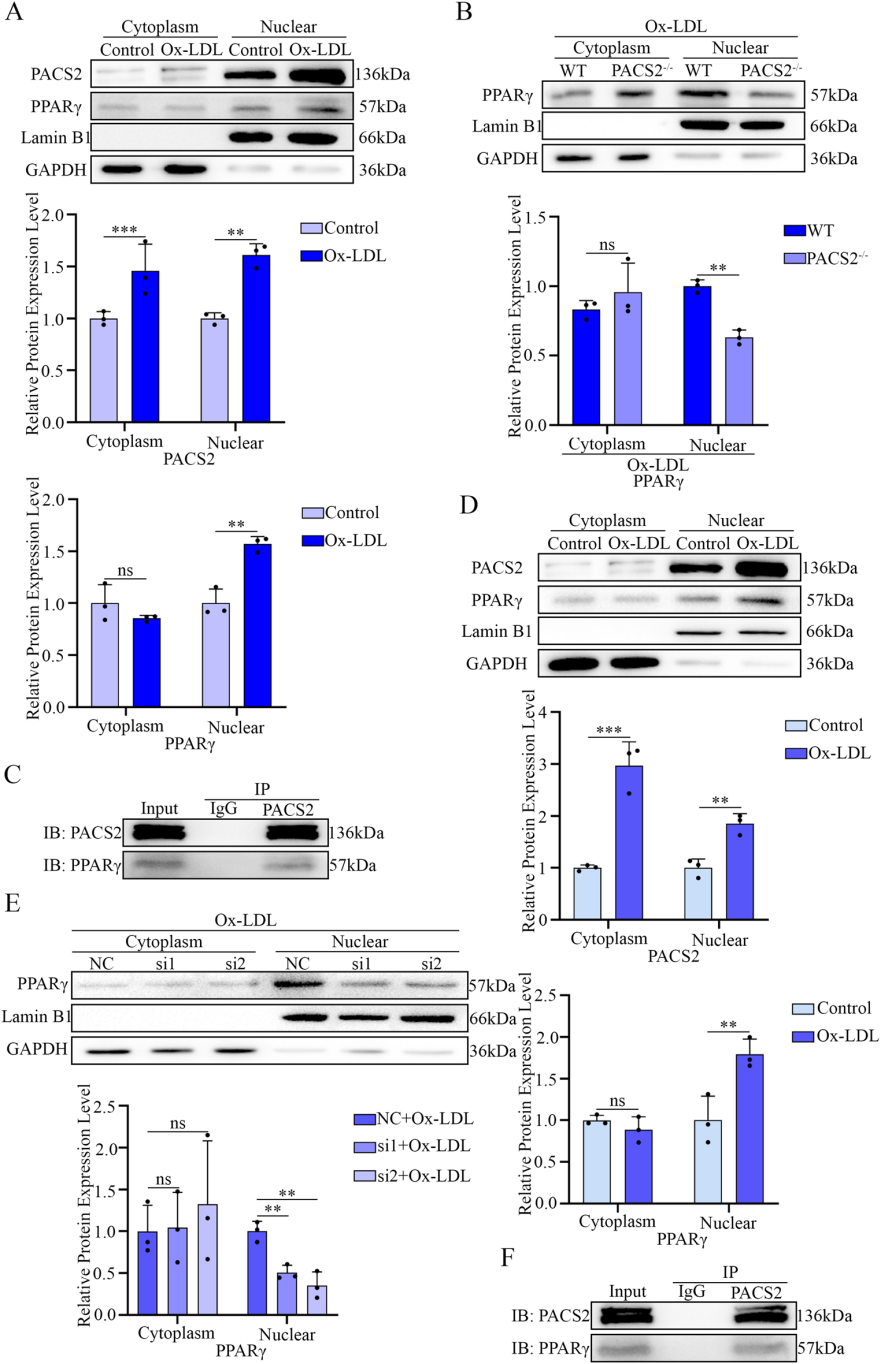
Inhibition of PACS2 reversed the Ox-LDL-induced nuclear translocation of PPARγ in macrophages. (**A**) Western blotting analyses and quantification of PACS2 and PPARγ expression in cytoplasmic fractions and nuclear fractions isolated from PMs exposed to Ox-LDL (*n* = 3). (**B**) Western blotting analysis and quantification of PPARγ expression in the cytoplasmic fractions and nuclear fractions of PMs exposed to Ox-LDL with or without PACS2 knockout (*n* = 3). (**C**) Coimmunoprecipitation analysis of PACS2 and PPARγ in PMs. (**D**) Western blotting analyses and quantification of PACS2 and PPARγ expression in cytoplasmic fractions and nuclear fractions isolated from RAW264.7 cells exposed to Ox-LDL (*n* = 3). (**E**) Western blotting analysis and quantification of PPARγ expression in the cytoplasmic fractions and nuclear fractions of RAW264.7 cells exposed to Ox-LDL with or without PACS2 knockout (*n* = 3). (**F**) Coimmunoprecipitation analysis of PACS2 and PPARγ in RAW264.7 cells

Emerging evidence suggests that PACS2 may regulate transcription factors [33]. Since PPARγ is a nuclear receptor and PACS2 contains a nuclear localization signal [34], we assessed whether PACS2 facilitates the nuclear translocation of PPARγ. Subcellular fractionation revealed increased nuclear accumulation of both PACS2 and PPARγ upon Ox-LDL stimulation (Fig. 12A and D). Conversely, PACS2 knockdown, which was induced via either genetic knockout (Fig. 12B) or siRNA-mediated silencing (Fig. 12E), reduced nuclear PPARγ levels. Coimmunoprecipitation confirmed the direct interaction between PACS2 and PPARγ in macrophages (Fig. 12C and F).

In summary, these results demonstrate that FTO inhibits the PPARγ-CD36/PLIN2 pathway by downregulating PACS2, which interacts with PPARγ and facilitates its nuclear translocation, thereby modulating lipid uptake and storage programs in macrophages.

### FTO alleviates atherosclerosis in a PACS2-dependent manner

**Fig. 13:**
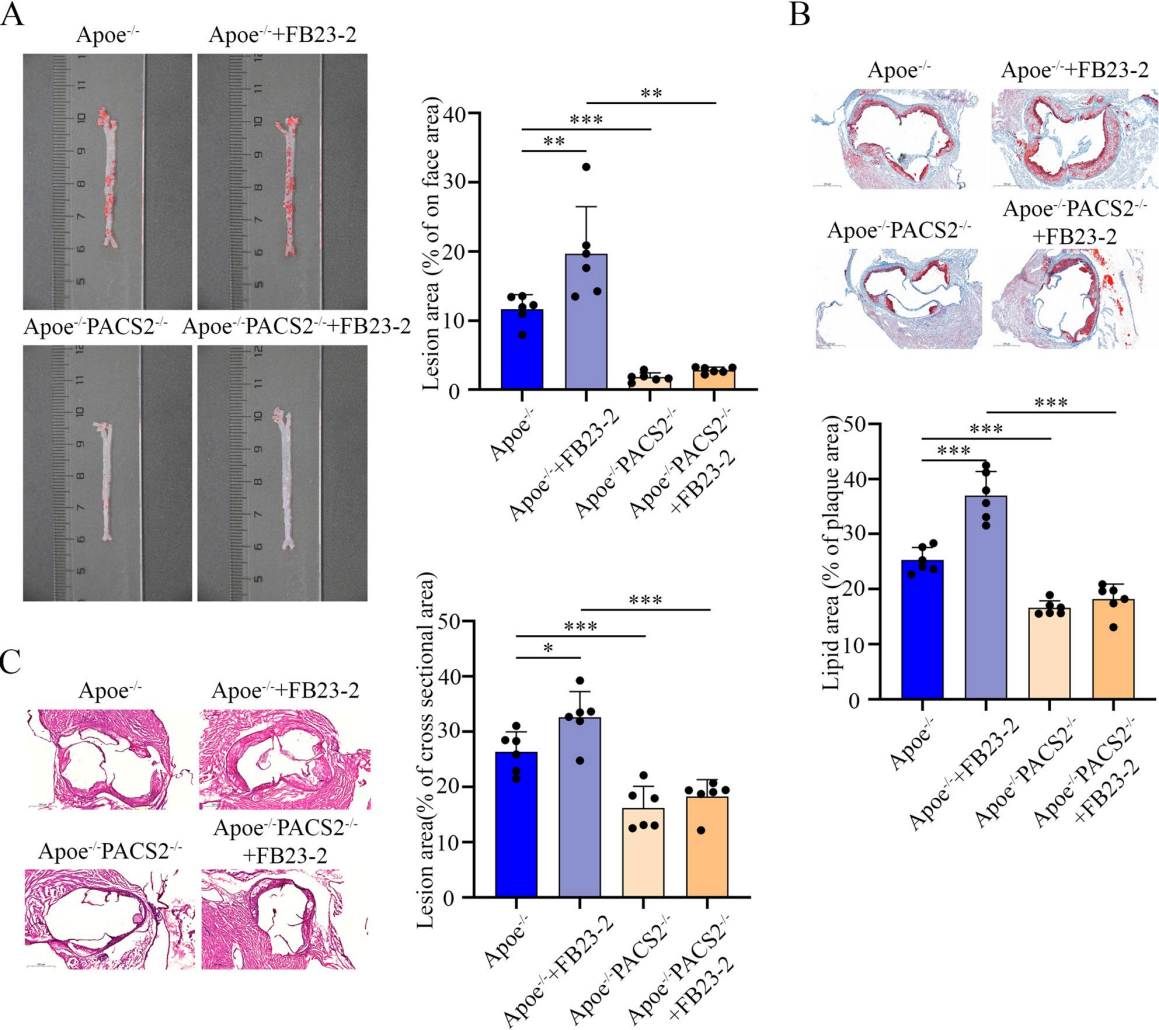
FTO-mediated mitigation of atherosclerosis is PACS2 dependent. (**A**) Representative Oil Red O staining images and quantification of atherosclerotic lesions in whole aortas from *ApoE*^*−/−*^ or *ApoE*^*−/−*^*PACS2*^*−/−*^ mice treated with or without FB23-2 (*n* = 6). (**B**) Representative Oil Red O staining images and quantification of atherosclerotic lesions in the aortic roots of *ApoE*^*−/−*^ or *ApoE*^*−/−*^*PACS2*^*−/−*^ mice treated with or without FB23-2 (*n* = 6). (**C**) Representative H&E staining images and quantification of atherosclerotic lesions in the aortic roots of *ApoE*^*−/−*^ or *ApoE*^*−/−*^*PACS2*^*−/−*^ mice treated with or without FB23-2 (*n* = 6)

To determine whether the atheroprotective effect of FTO depends on PACS2, we utilized *ApoE*^*−/−*^*PACS2*^*−/−*^ mice, a model that has been previously established by our group [18]. These mice, along with *ApoE*^*−/−*^ controls, were fed an HFD and treated with the FTO inhibitor FB23-2. En face analysis of aortas revealed that PACS2 knockout not only attenuated atherosclerosis progression but also largely abolished the exacerbation of lesion development induced by FB23-2 (Fig. 13A). Consistent with these findings, histological examination of aortic root sections revealed that PACS2 deficiency significantly mitigated the FB23-2-induced increase in plaque burden, as demonstrated by H&E and Oil Red O staining (Fig. 13B and C).

These results demonstrate that the protective effect of FTO against atherosclerosis is mediated through the downregulation of PACS2.

## Discussion

Macrophages play a pivotal role in the progression of atherosclerosis, particularly through foam cell formation and phenotypic plasticity [35, 36]. An understanding of the regulatory mechanisms underlying macrophage foaming is essential for the development of novel therapeutic strategies. Ox-LDL represents a major risk factor for atherogenesis [37]. In this study, we demonstrated that FTO expression is downregulated in macrophages under atherosclerotic conditions, thus leading to altered intracellular m^6^A methylation levels. FTO, the first identified m^6^A demethylase, is a critical element in epitranscriptomic research. Previous studies have reported that FTO mitigates hepatic ischemia/reperfusion injury by downregulating ACSL4 and TFRC [28] and counteracts cellular senescence by stabilizing MIS12 in human embryonic stem cells [38]. Here, we revealed a novel mechanism by which reduced FTO expression in macrophages increases PACS2 abundance through the m^6^A methylation of *PACS2* mRNA, thereby promoting lipid uptake and deposition and exacerbating foam cell formation.

PACS2, which is located on the mitochondria-associated endoplasmic reticulum membrane, remains incompletely understood in the context of atherosclerosis. Our earlier research revealed that PACS2 promotes foam cell formation by enhancing lipid uptake [18]. In this study, we further demonstrated that FTO erases m^6^A modifications on *PACS2* mRNA and facilitates its recognition by the m^6^A reader YTHDF2, thus leading to decreased mRNA stability and suppressed PACS2 expression. This mechanism effectively inhibited Ox-LDL-induced lipid uptake and deposition in macrophages, and these effects were reversed upon PACS2 overexpression.

Peroxisome proliferator-activated receptors (PPARs), including PPAR-α, PPAR-δ, and PPARγ, are ligand-activated transcription factors that are implicated in atherosclerosis, inflammation, and hypertension [39, 40]. In particular, PPARγ activation regulates macrophage lipid metabolism [41].

Although PPARγ modulation has been extensively studied, its regulation via m^6^A-dependent mRNA metabolism remains unexplored. We demonstrate for the first time that macrophage-specific FTO overexpression suppresses Ox-LDL-induced PPARγ activation by downregulating PACS2 expression. Consequently, PPARγ inhibition reduced both lipid uptake via CD36 downregulation and lipid deposition via PLIN2 suppression, both of which were reversed by PACS2 overexpression.

Emerging evidence suggests that PACS2 can influence transcription factors [33]. Our data indicate that PACS2 knockout or knockdown attenuates Ox-LDL-induced PPARγ nuclear translocation. PACS2 is a membrane transporter protein [34]. As a multidomain sorting protein, PACS2 contains a cargo-binding region, a disordered intermediate region, and a carboxy-terminal region [34]. The cargo-binding region can bind to cargo proteins containing acidic clusters and transport these cargo proteins to corresponding organelles [42–44]. In addition, the disordered intermediate region of PACS2 contains a nuclear localization signal [33], thus suggesting a potential role in nucleocytoplasmic trafficking. In the current study, we revealed that PPARγ can be recognized by PACS2. Using a CO-IP assay, we also demonstrated that PACS2 could bind to PPARγ. These findings suggested that PACS2 may promote macrophage foaming by maintaining lipid uptake and deposition through interactions with PPARγ and by mediating its nuclear translocation. Moreover, FTO can inhibit the expression of PACS2 by erasing the m^6^A modification on *PACS2* mRNA, which can block this adverse process.

Interestingly, although our previous work demonstrated that PACS2 deficiency reduces circulating lipid levels in atherosclerotic mice, in the present study, macrophage-specific FTO overexpression did not significantly alter systemic lipid profiles. This discrepancy may reflect differences in regulatory hierarchy and cell-type specificity. As an upstream epitranscriptomic regulator, FTO primarily modulates macrophage lipid handling and foam cell formation within atherosclerotic lesions, whereas PACS2 may exert broader effects on systemic lipid metabolism. These findings suggest that the proatherogenic role of the FTO–PACS2 axis is driven predominantly by local plaque macrophage dysfunction rather than whole-body lipid alterations.

**Fig. 14:**
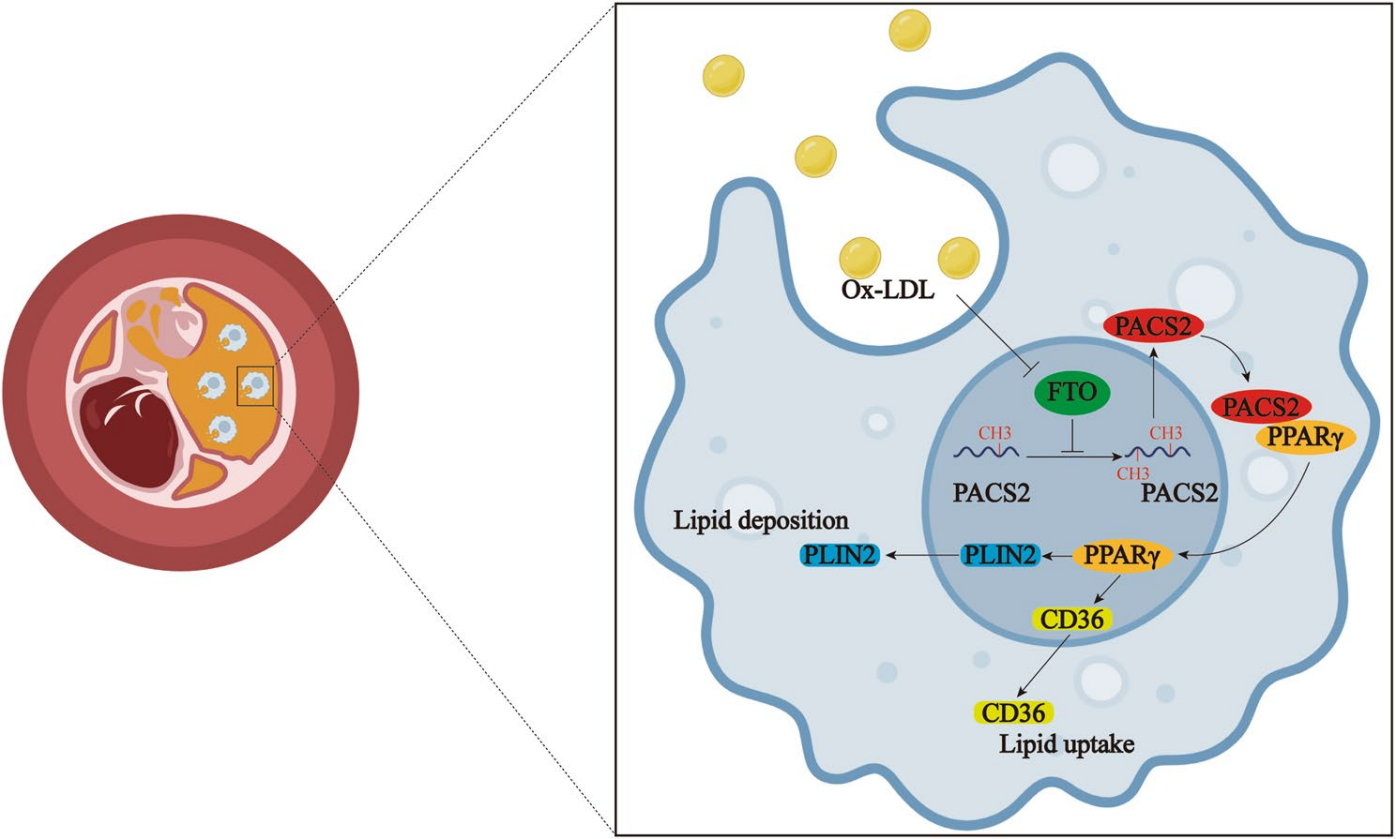
Schematic diagram depicting how FTO deficiency in macrophages triggers lipid uptake and deposition during atherosclerosis by increasing PACS2 m^6^A modification

In summary, our study demonstrates that the enhancement of FTO activity attenuates atherosclerosis by downregulating PACS2, which is a key driver of macrophage foam cell formation. FTO negatively regulates the PACS2-PPARγ-CD36/PLIN2 axis through YTHDF2-dependent m^6^A-modified *PACS2* mRNA (Fig. 14). These findings provide new insights into the epitranscriptional regulation of macrophage lipid metabolism and identify FTO as a potential therapeutic target for atherosclerotic cardiovascular disease. Further clinical studies are warranted to evaluate the translational potential of the targeting of FTO in mitigating macrophage foaming and plaque progression.

Despite these advances, several limitations of the present study should be acknowledged. First, validation of FTO, PACS2, and downstream signaling molecules in human atherosclerotic plaque tissues was not performed, and their clinical relevance requires further confirmation, which is an important direction for future translational investigation. Second, macrophages within atherosclerotic lesions are heterogeneous in origin; subset-specific markers and lineage-tracing approaches were not employed in this study to distinguish tissue-resident from monocyte-derived macrophages. In addition, the present work focused predominantly on macrophage-driven mechanisms without assessing the contribution of other vascular cell types, such as endothelial cells or vascular smooth muscle cells. Finally, although our findings highlight FTO as a potential therapeutic target, the translational feasibility and safety of targeting the FTO–PACS2 axis warrant further investigation in future preclinical and clinical studies.

## Supplementary Information

Below is the link to the electronic supplementary material.

Supplementary Material 1: Supplementary Fig. 1. (A) Body weight of the mice treated with AAV-Null or AAV-FTO (n = 6). (B) Levels of triglycerides, cholesterol, high-density lipoprotein, and low-density lipoprotein in the mice treated with AAV-Null or AAV-FTO (n = 6).

## Data Availability

The datasets used and/or analyzed during the current study are available from the corresponding author on reasonable request.
