# Supplementary material for: Macrophage FTO deficiency accelerates atherosclerosis via PACS2-mediated activation of the PPARγ lipid signaling pathway
Source: J Transl Med. 2026 Mar 31;24:644. doi: 10.1186/s12967-026-08076-3 (PMC13154680; doi:10.1186/s12967-026-08076-3)

Figure 1

Figure 1B: FTO

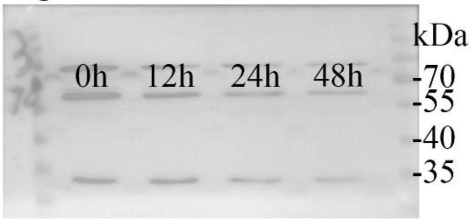

Figure 1B: FTO-GAPDH

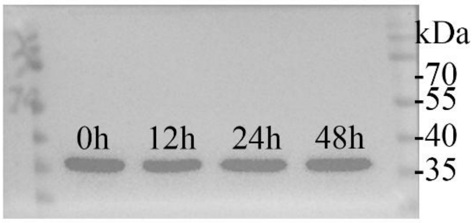

Figure 1D: FTO

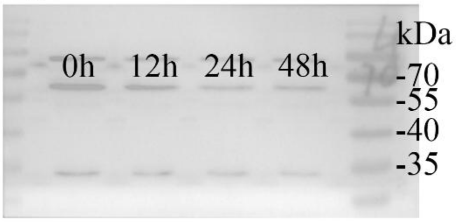

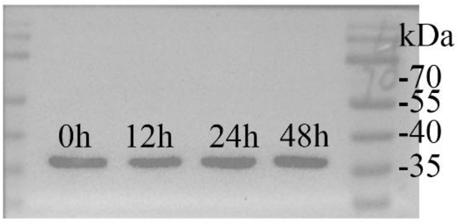

Figure 2

Figure 2B: FTO

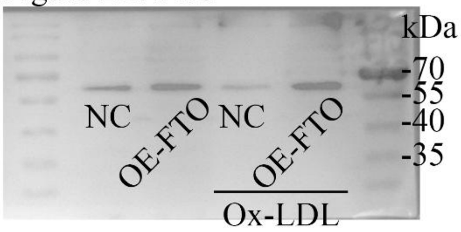

Figure 2B: FTO-GAPDH

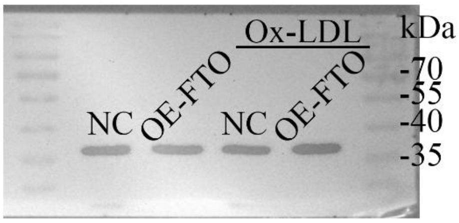

Figure 2D: FTO

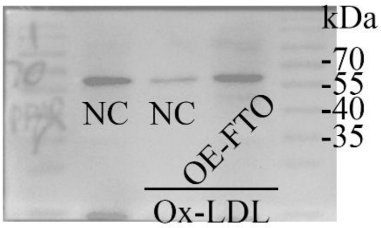

Figure 2D: FTO-GAPDH

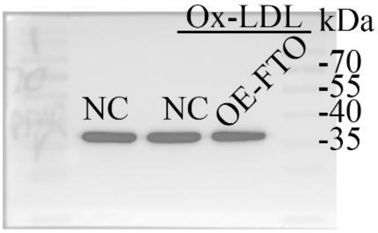

Figure 4

Figure 4B: FTO

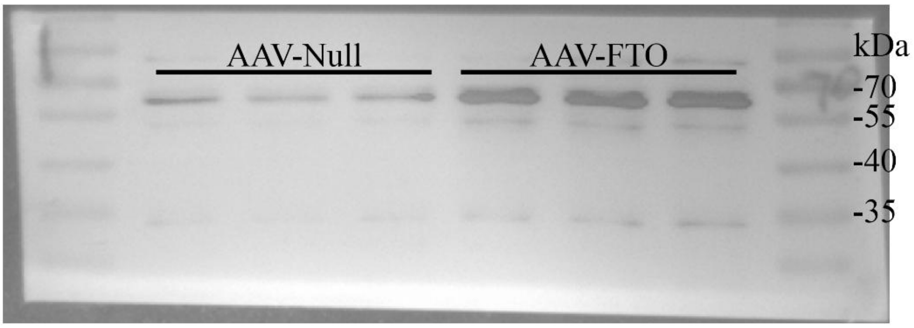

Figure 4B: FTO-GAPDH

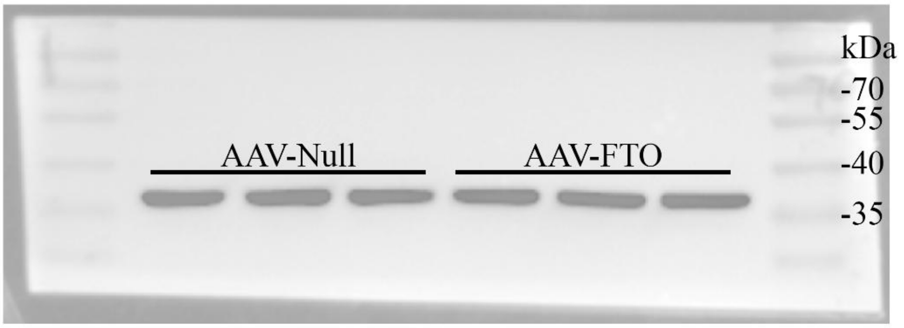

Figure 5

Figure 5C: PACS2

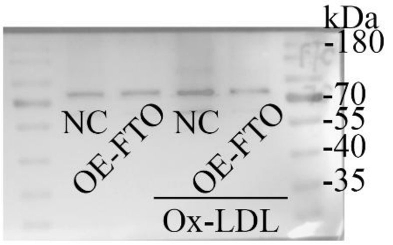

Figure 5C: PACS2-GAPDH

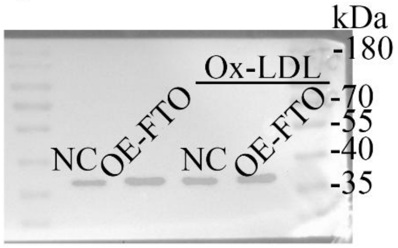

Figure 5F: PACS2

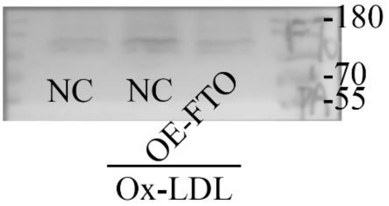

Figure 5F: PACS2-GAPDH

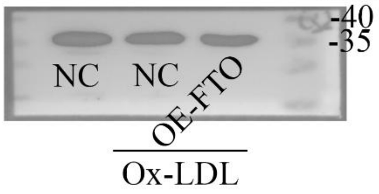

Figure 7

Figure 7I: YTHDF2

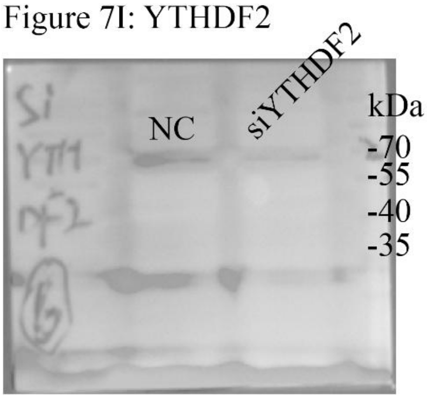

Figure 7I: YTHDF2-GAPDH

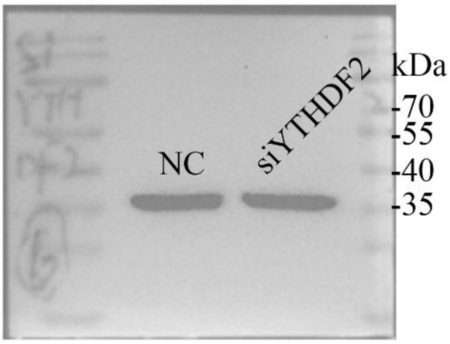

Figure 7I: PACS2

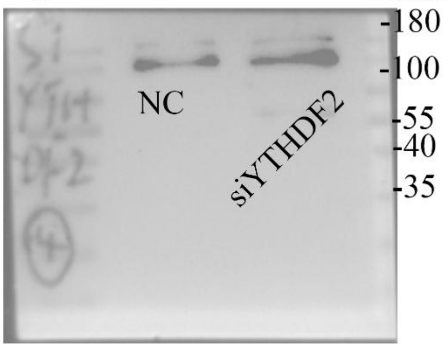

Figure 7J: PACS2-GAPDH

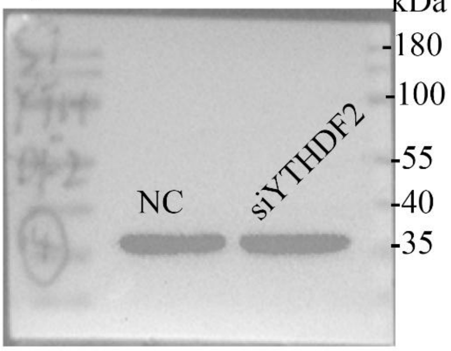

Figure 7J: YTHDF2

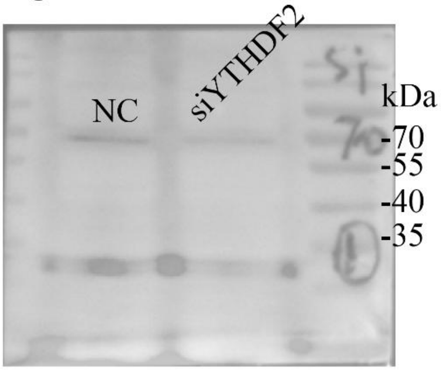

Figure 7J: YTHDF2-GAPDH

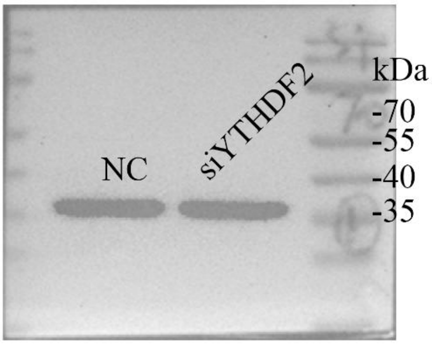

Figure 7J: PACS2

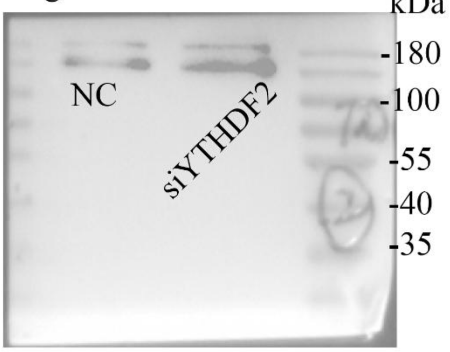

Figure 7J: PACS2-GAPDH

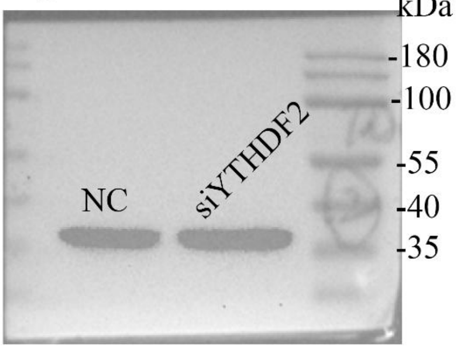

Figure 8

Figure 8C: FTO

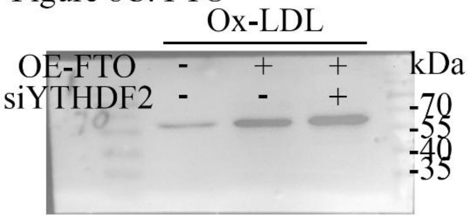

Figure 8C: FTO-GAPDH

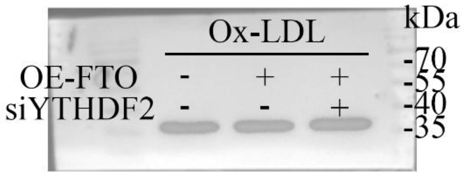

Figure 8C: YTHDF2

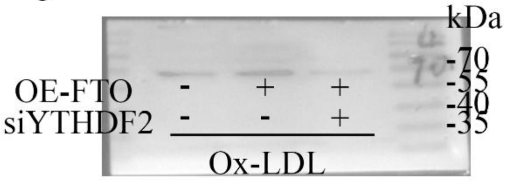

Figure 8C: YTHDF2-GAPDH

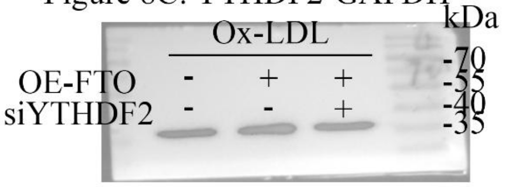

Figure 8C: PACS2

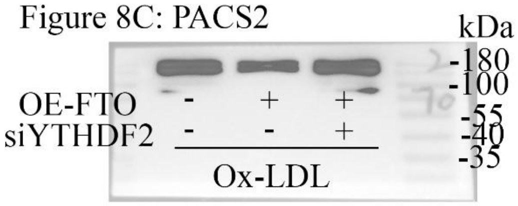

Figure 8C: PACS2-GAPDH

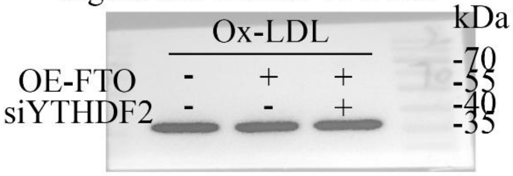

Figure 8D: FTO

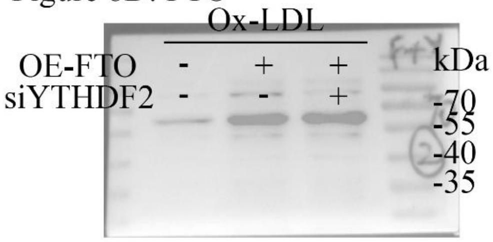

Figure 8D: FTO-GAPDH

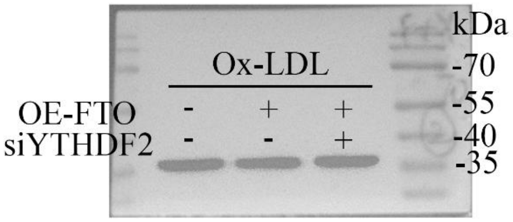

Figure 8D: YTHDF2

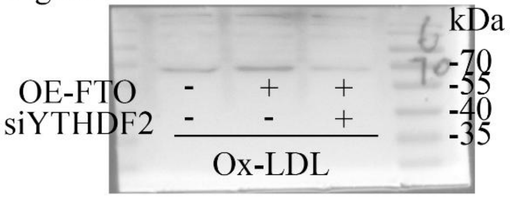

Figure 8D: YTHDF2-GAPDH

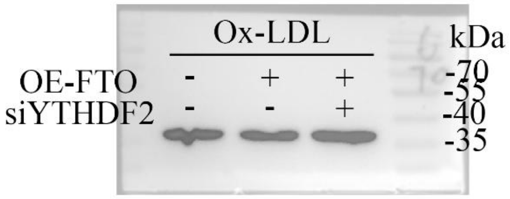

Figure 8D: PACS2

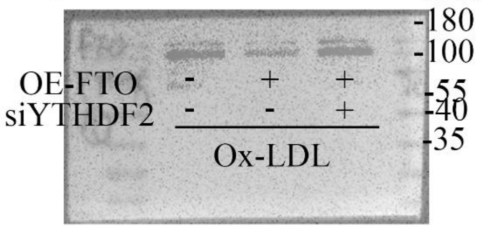

Figure 8D: PACS2-GAPDH

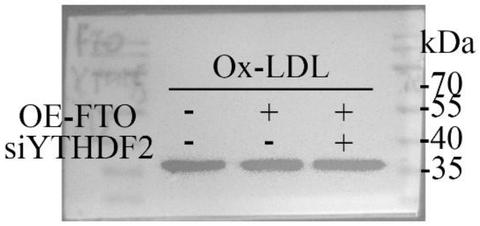

Figure 10

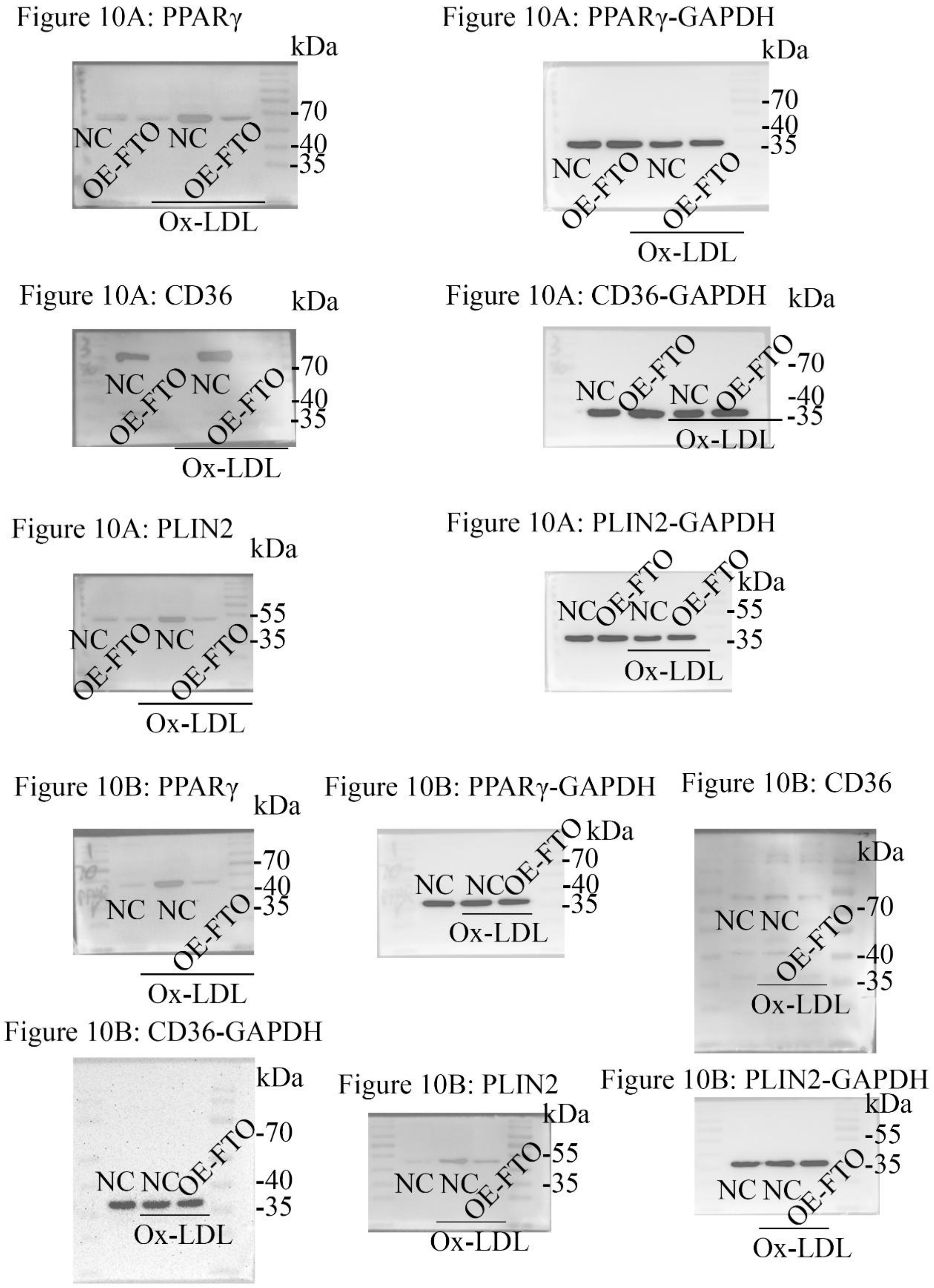

Figure 10A: PPARγ
kDa
-70
-40
-35
NC
OE-FTO
NC
OE-FTO
Ox-LDL
Figure 10A: PPARγ-GAPDH
kDa
-70
-40
-35
NC
OE-FTO
NC
OE-FTO
Ox-LDL
Figure 10A: CD36
kDa
-70
-40
-35
NC
OE-FTO
NC
OE-FTO
Ox-LDL
Figure 10A: CD36-GAPDH
kDa
-70
-40
-35
NC
OE-FTO
NC
OE-FTO
Ox-LDL
Figure 10A: PLIN2
kDa
-55
-35
NC
OE-FTO
NC
OE-FTO
Ox-LDL
Figure 10A: PLIN2-GAPDH
kDa
-55
-35
NC
OE-FTO
NC
OE-FTO
Ox-LDL
Figure 10B: PPARγ
kDa
-70
-40
-35
NC
NC
OE-FTO
Ox-LDL
Figure 10B: PPARγ-GAPDH
kDa
-70
-40
-35
NC
NC
OE-FTO
Ox-LDL
Figure 10B: CD36
kDa
-70
-40
-35
NC
NC
OE-FTO
Ox-LDL
Figure 10B: CD36-GAPDH
kDa
-70
-40
-35
NC
NC
OE-FTO
Ox-LDL
Figure 10B: PLIN2
kDa
-55
-35
NC
NC
OE-FTO
Ox-LDL
Figure 10B: PLIN2-GAPDH
kDa
-55
-35
NC
NC
OE-FTO
Ox-LDL

Figure 11

Figure 11A: FTO

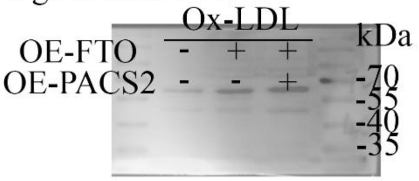

Figure 11A: FTO-GAPDH

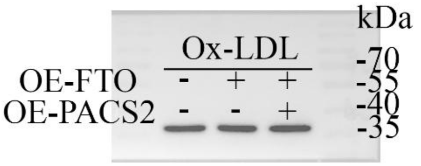

Figure 11A: PACS2

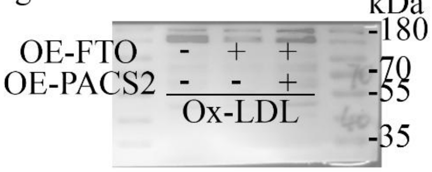

Figure 11A: PACS2-GAPDH

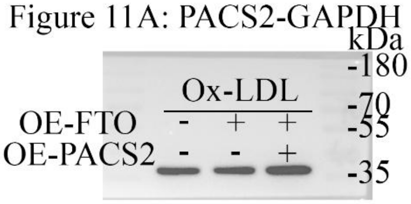

Figure 11A: PPARγ

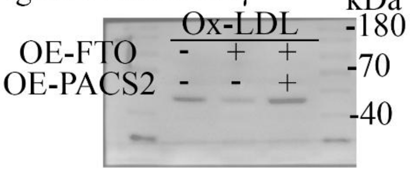

Figure 11A: PPARγ-GAPDH

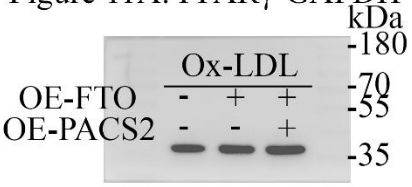

Figure 11A: CD36

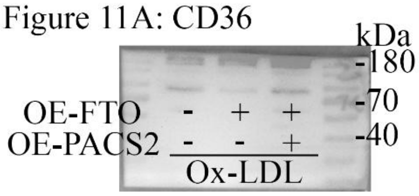

Figure 11A: CD36-GAPDH

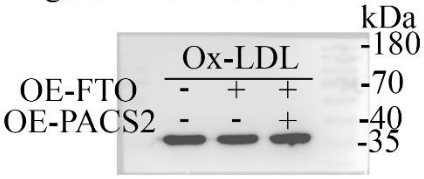

Figure 11A: PLIN2

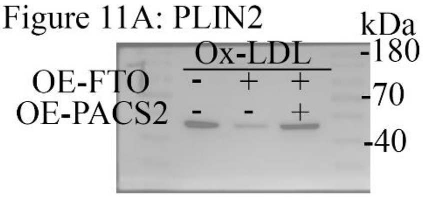

Figure 11A: PLIN2-GAPDH

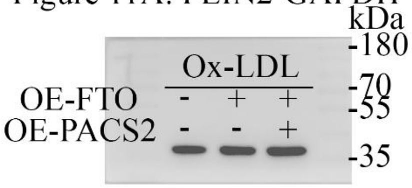

Figure 11B: FTO

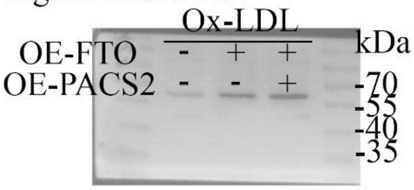

Figure 11B: FTO-GAPDH

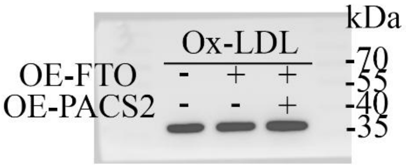

Figure 11B: PACS2

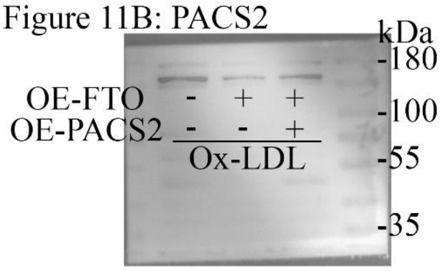

Figure 11B: PACS2-GAPDH

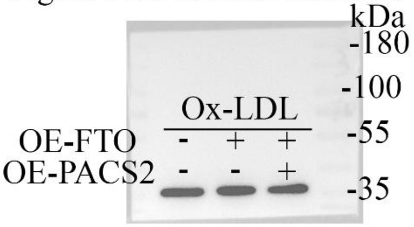

Figure 11B: PPARγ

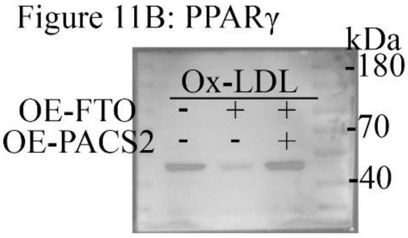

Figure 11B: PPARγ-GAPDH

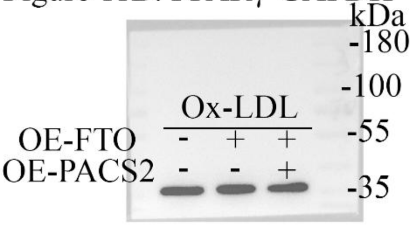

Figure 11B: CD36

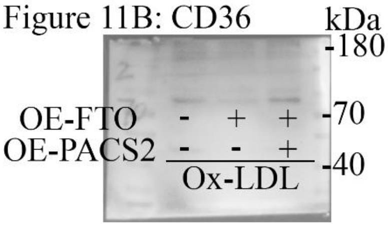

Figure 11B: CD36-GAPDH

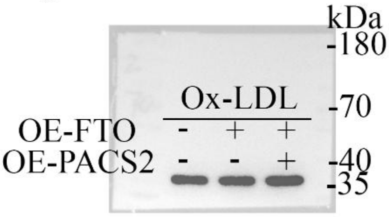

Figure 11B: PLIN2

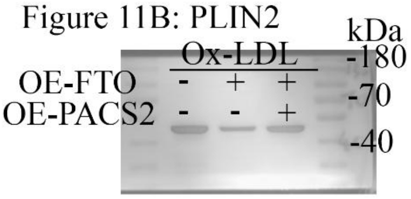

Figure 11B: PLIN2-GAPDH

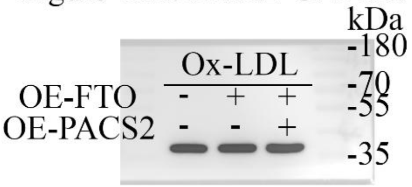

Figure 12

Figure 12A: PACS2

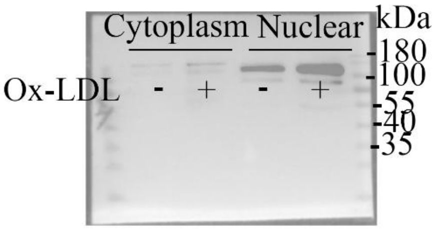

Figure 12A: PPARγ

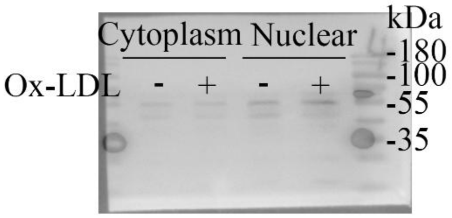

Figure 12A: LaminB1

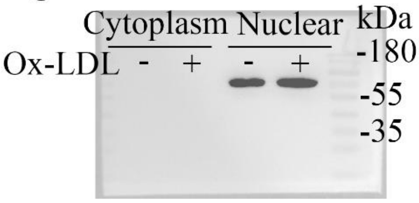

Figure 12A: GAPDH

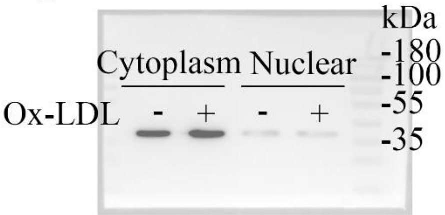

Figure 12B: PPARγ

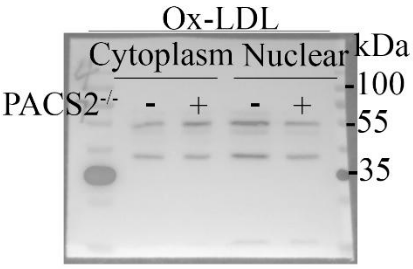

Figure 12B: LaminB1

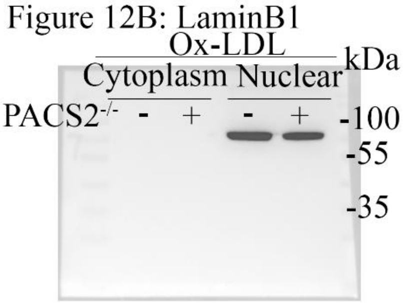

Figure 12B: GAPDH

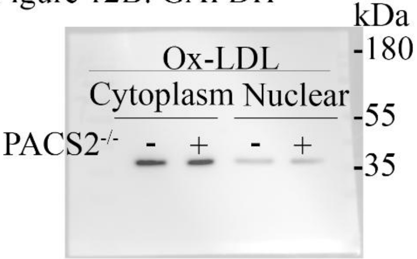

Figure 12C: PACS2

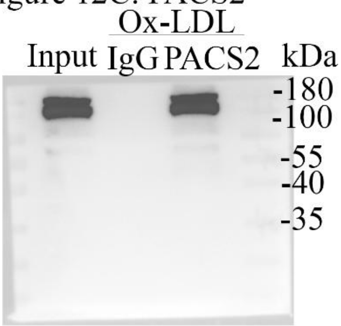

Figure 12C: PPARγ

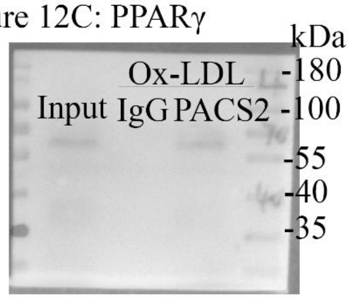

Figure 12D: PACS2

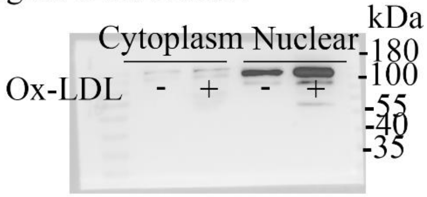

Figure 12D: PPARγ

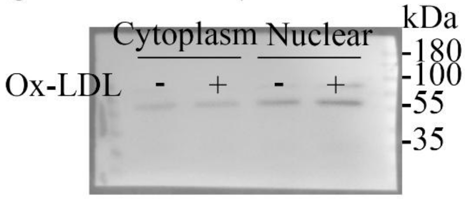

Figure 12D: LaminB1

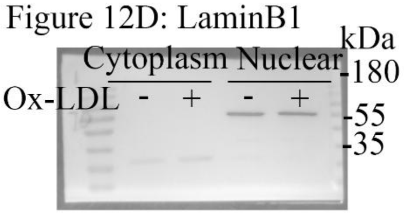

Figure 12D: GAPDH

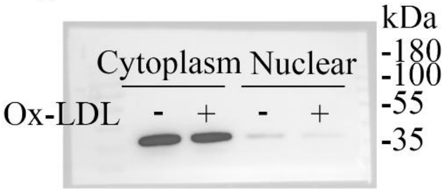

Figure 12E: PPARγ

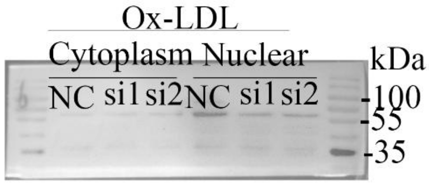

Figure 12E: LaminB1

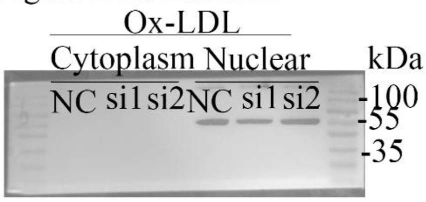

Figure 12E: GAPDH

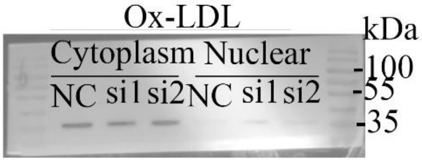

Figure 12F: PACS2

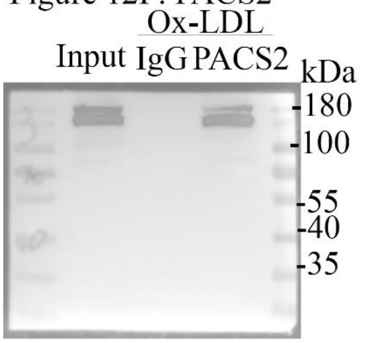

Figure 12F: PPARγ

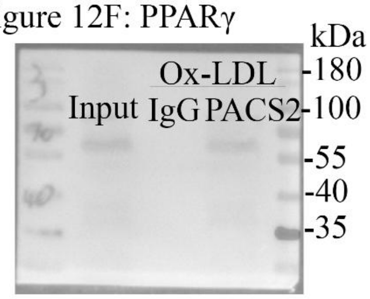

Supplement: Supplementary file 1 — Supplementary Material 1: Supplementary Fig. 1. (A) Body weight of the mice treated with AAV-Null or AAV-FTO (n = 6). (B) Levels of triglycerides, cholesterol, high-density lipoprotein, and low-density lipoprotein in the mice treated with AAV-Null or AAV-FTO (n = 6). [file 12967_2026_8076_MOESM1_ESM.pdf]
